# Confidently Uncertain: Probabilistic Machine Learning to Predict Soil Biotransformation Half-Lives

**DOI:** 10.1021/acs.est.6c03516

**Published:** 2026-04-02

**Authors:** Moritz Salz, José Andrés Cordero Solano, Kathrin Fenner, Jasmin Hafner

**Affiliations:** † Department of Environmental Chemistry, Eawag, Dübendorf 8600, Switzerland; ‡ Department of Chemistry, University of Zurich, Zürich 8006, Switzerland

**Keywords:** environmental persistence, soil biotransformation half-lives, Gaussian process regression, biodegradation, probabilistic modeling, pesticides

## Abstract

Predicting environmental persistence of chemicals from molecular structure is an open challenge, yet indispensable in regulatory screenings for potentially harmful substances and to advance the development of safe-and-sustainable-by-design chemicals. Limited availability of biotransformation half-life data makes persistence prediction difficult, and models typically struggle to generalize beyond their training data. Therefore, reliable estimates of prediction confidence are key. Here, we propose a probabilistic model for the prediction of soil biotransformation half-lives. A Gaussian Process Regressor was trained on 867 mean pesticide half-lives with data uncertainty estimates. Instead of single half-life values, our model predicts well-calibrated probability distributions that can be used to calculate a compound’s probability of being persistent. Although the overall model performance remains moderate, the predictions are reliable when the confidence in the prediction is high. We applied our model to pesticide transformation products with unknown half-lives, and to a database of globally marketed chemicals. We show that our model is able to identify chemicals that are known, or suspected to be, persistent in the environment. The model is available as an online app (https://pepper-app.streamlit.app/) and as a Python library (pepper-lab) to meet diverse user needs.

## Graphical abstract

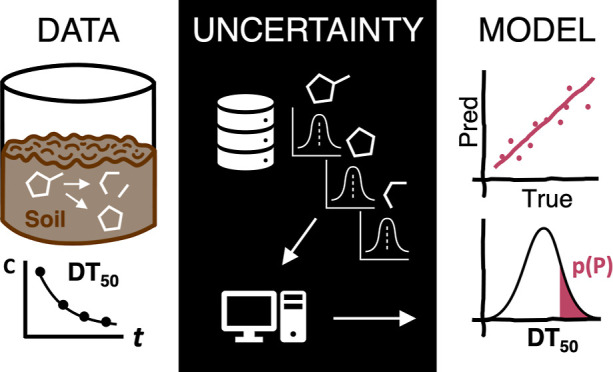

## Introduction

Anthropogenic chemicals that persist in the environment pose a risk to ecosystem integrity and to human health. As reversing micropollutant contamination is technically challenging, energy-intensive, and costly,^1^ persistence (P) assessment is part of chemical regulation,^2^ and it has been argued that persistence alone should suffice as grounds for regulatory action.^3^ In regulatory persistence assessment, screening tests (e.g., ready biodegradability) are considered to detect easily degraded substances, yet, for all others, simulation tests are required for persistence assessment, i.e., to obtain primary biotransformation half-lives (DT_50_) and information on transformation products in different environmental systems (e.g., soil, water-sediment systems, surface water).^4^ As such tests are costly and time-consuming, the 2025 European Chemical Agency (ECHA) report identifies the development of screening methods for persistence assessment as a key area of regulatory challenge.^5^ Hence, new in vitro or in silico approaches are needed to screen long lists of chemicals for persistence, both for regulatory purposes and to meet the Safe-and-Sustainable-by-Design (SSbD) objectives in industrial research and development.

In silico methods, particularly Quantitative Structure–Activity Relationship (QSAR) models, offer a cost-effective approach to estimate persistence. QSAR models infer persistence from molecular structure under the assumption that structurally similar chemicals exhibit similar environmental behaviors.^6^ Models have been developed to predict ready biodegradability (RB),^7–11^ as data for this end point are more abundant than for simulation studies. However, RB testing results in a yes/no answer and is of limited use to distinguish low to moderately persistent chemicals from very persistent chemicals. Calculating primary biotransformation half-life (DT_50_) from experimentally observed concentration–time series can provide a better resolution of a molecule’s persistence, with the limitation that it only describes the kinetics of the first biotransformation step. In addition, knowledge on the formed transformation products and their DT_50_ is needed to fully understand a molecule’s persistence. Hence, predicting a representative DT_50_ from structure is a key step in persistence prediction, but existing models either focus on narrow classes of chemicals (e.g., Opera BioHL for hydrocarbons^6^), or mostly rely on expert estimations rather than experimental data (EPISuite Biowin,^12^ VEGA^13^). One major bottleneck in predicting primary biotransformation half-lives is the lack of sufficiently big data sets,^14^ which is aggravated by the high experimental variability observed in the outcomes of biodegradation simulation tests. Reasons for the variability in reported DT_50_ are differences in the sampled inocula (e.g., testing in different soils), experimental procedures, and analytical methods. Additional variability may also arise from the use of different kinetic assumptions to derive DT_50_ values from concentration–time series. Moreover, for some compounds, the low number of experimental replicates leads to low confidence in the calculated mean DT_50_ of a substance.^15^

In this work, we build machine-learning models that predict soil primary biotransformation half-lives with associated prediction confidence from molecular structure, aiming to provide a reliable persistence prediction tool for screening long lists of untested substances in chemical regulation, and that could also be integrated into SSbD frameworks (e.g., PARC^16^). Our training data set comprises 6309 biotransformation half-lives for 867 pesticides and pesticide transformation products (TPs) obtained from simulation studies in soil according to the Organization for Economic Co-operation and Development (OECD) 307 guideline.^17^ As our objective is to predict a representative half-life for a compound under a variety of conditions, we quantify experimental variability as well as the confidence in the estimated mean DT_50_ using Bayesian inference.^15^ The Bayesian-inferred mean DT_50_ values and their uncertainty constitute the input for our probabilistic modeling approach, which considers data uncertainty in the training data and explicitly quantifies prediction uncertainty in predicted half-lives. Unlike classical QSAR methods that yield only single-point estimates, our methodology produces full predictive distributions and thereby explicitly quantifies both aleatoric (data-inherent) and epistemic (model-based) uncertainty. We test combinations of molecular descriptors and algorithms to optimize model performance, and we assess the reliability of the predicted uncertainty metrics. We then apply the final model to estimate DT_50_ for pesticide transformation products lacking kinetic information and to the whole space of marketed chemicals.

## Methods

### Workflow

The path toward probabilistic models consists of two major steps: (i) obtaining a characteristic half-life distribution from several reported DT_50_ values from soil biodegradation studies performed in different soils and hence under different experimental conditions (Figure 1a). (ii) Training machine-learning models on characteristic half-life distributions and structural descriptors to predict half-lives with uncertainty (Figure 1b). The steps involved in this process are detailed in the following subsections.

**Figure 1:**
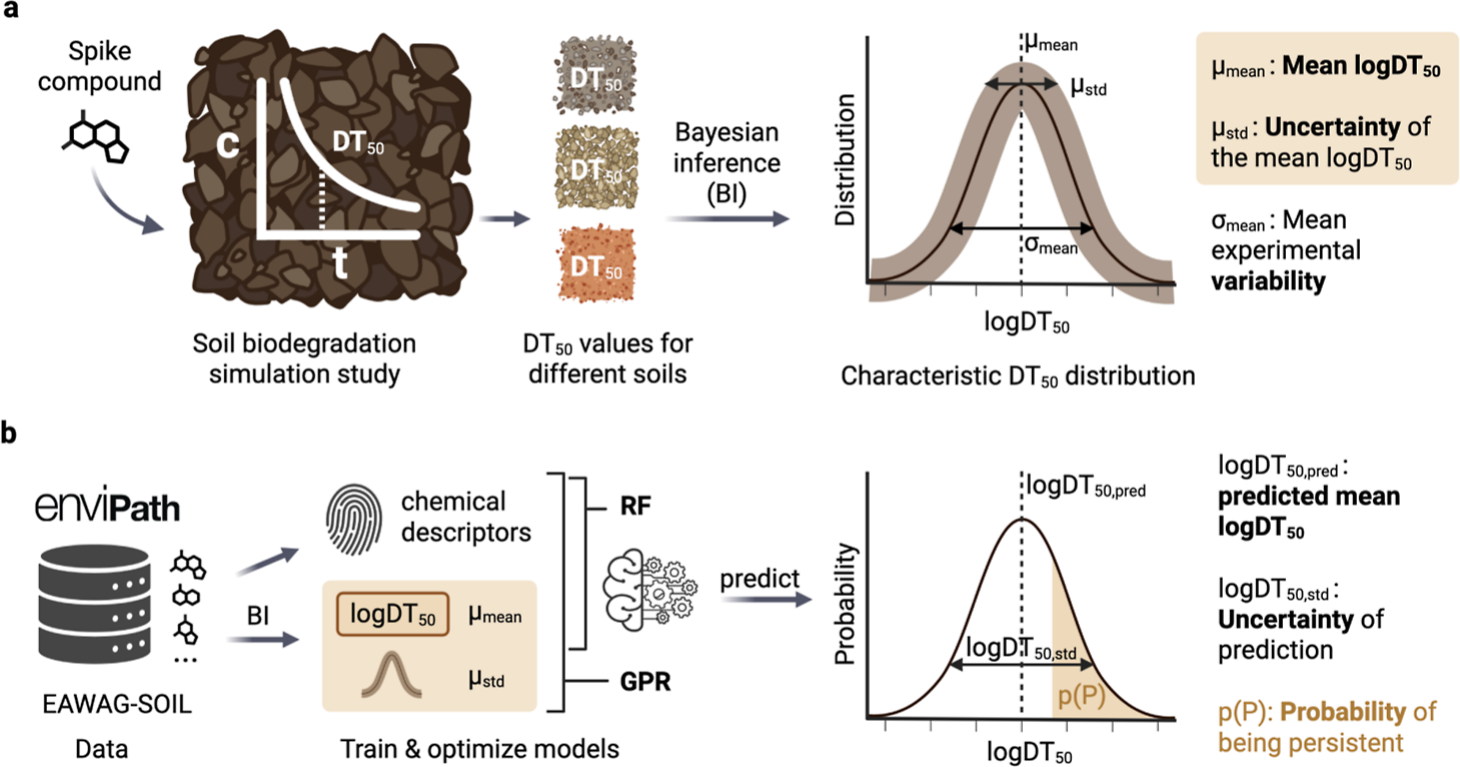
Workflow toward probabilistic biotransformation half-life predictions. (a) A characteristic DT_50_ distribution is obtained from experimental observations under varying soil conditions. (b) Two different models are built using biodegradation data from the EAWAG-SOIL package in enviPath. The output of the models is the predicted target variable (logDT_50,mean_) plus the uncertainty of the prediction as a standard deviation (logDT_50,std_). RF: Random Forest regressor, GPR: Gaussian Process Regressor. Figure created with biorender.com.

### Data Curation and Bayesian Inference

The soil biotransformation half-lives were downloaded from the EAWAG-SOIL data package (https://envipath.org/package/5882df9c-dae1-4d80-a40e-db4724271456) on enviPath^18^ on 7 April 2025. The package contains pesticide biotransformation pathways and reported half-lives observed in soil biodegradation simulation experiments mostly following the OECD 307 guideline.^19^ Inorganic compounds and composite mixtures were excluded, and stereochemical information was removed from all substances as stereospecific half-life prediction is beyond the scope of this work. To encapsulate the high variability in observed DT_50_ and to get meaningful mean estimates for compounds including censored values (i.e., <, > values), distributions of log10 experimental half-lives log DT_50_ were inferred per compound using a Bayesian inference framework as rationalized and described in Hafner et al. (2023)^15^ (Figure 1a, Section S1.1).

Using the inferred distributions, we quantified the probability that the mean soil half-life of a compound exceeds regulatory persistence thresholds defined under REACH (<120 days for nonpersistent (nP), >120 days for persistent (P), >180 days for very persistent (vP)). For any threshold *T*(P, vP), the probability of exceeding *T* is obtained via

1 $$p\left(\log DT_{50}\geq \log T\right)=1-\Phi \left(\frac{\log T-\mu_{mean}}{\mu_{std}}\right)$$

where Φ is the cumulative distribution function of the standard normal distribution. Overall, the probability of a compound to be persistent was calculated as *p*(P) = *p*(log DT_50_ ≥ 120) and to be very-persistent as *p*(vP) = *p*(log DT_50_ ≥ 180). The probability for a compound to be nP then is given by 1 – *p*(P). An example calculation for an example compound is visualized in Figure S4.

### Modeling

To address the limitations of classical QSAR models in quantifying predictive uncertainty, especially when applied to small, noisy data sets, we focused on two modeling approaches: Gaussian Process Regression (GPR) and Random Forest (RF) as a baseline (Figure 1b). For both models, the Bayesian inferred mean half-lives were used as target values.

GPR offers a fully probabilistic framework. As GPR provides a Gaussian probability distribution for each predicted value (rather than just a single point estimate), the GPR model trained on soil half-lives renders both a mean predicted half-life (logDT_50,pred_) and an associated uncertainty (logDT_50,std_). In areas of the chemical space with a higher coverage of training data, a GPR model is more confident (smaller uncertainties) than in areas of sparse data. A GPR is defined by a prior assumption about the smoothness of the underlying relationship between encoded molecular structures and half-lives. This prior is characterized through the kernel (i.e., covariance function), which quantifies how close any two compounds are in the feature space.^20^ Furthermore, incorporating target variable uncertainty μ_std_ into the fitting process as a heteroscedastic noise term α = μ_std_^2^ ^21^ allows model training to focus on compounds with confident half-lives, while avoiding overfitting to data with high uncertainty.

A RF regressor was used as a baseline model for comparison. We followed the same setup as for a previously reported RF model for Wastewater Treatment Plant (WWTP) Breakthrough prediction,^22^ using the empirical standard deviation across individual trees as a proxy for the predictive uncertainty.

### Features & Hyperparameters

To translate chemical structures into numerical features, we tested different fingerprints and descriptor sets. In general, fingerprints represent molecular structures as high-dimensional binary vectors indicating the presence or absence of substructures, whereas descriptors encode defined molecular properties as continuous values. The fingerprints representations included MACCS keys, capturing the presence or absence of 166 predefined substructures,^23^ triggered biotransformation rules (ePFP),^22^ representing functional groups that are known to undergo specific enzymatic transformation based on the enviPath database,^18^ Avalon fingerprints, which are hashed substructure-based representations encoding atom-bond patterns,^24^ and RDKit path-based fingerprints (2048-bit), capturing linear topological substructures. The descriptor representations included PaDEL descriptors, providing a comprehensive set of 1D, 2D and 3D molecular properties including constitutional, topological, and physicochemical characteristics,^25^ as well as RDKit descriptors.^26^ Descriptors and fingerprints were calculated for all compounds. Features that could not be calculated for all compounds were removed, and all features were subsequently min-max-scaled. The features were then preselected with a variance threshold of 0.02 to retain only informative features. To reduce redundancy among features, pairwise Spearman correlations were computed and converted to a distance matrix (1 – |*r*|), which was subjected to hierarchical clustering. Features grouped into highly correlated clusters (0.01 distance, i.e., |*r*| ≥ 0.99) were considered redundant, and one representative feature per cluster was retained for modeling. For both RF and GPR models, each fingerprint or descriptor set was evaluated separately and in combination to identify the best performing representations (Table S2). To test whether dimensionality reduction improved performance, we applied Principal Component Analysis (PCA) on the GPR models (Section S2, Table S3).

### Model Evaluation

Model evaluation and hyperparameter selection were performed using 5-fold nested cross-validation, with hyperparameters optimized within the inner folds and model performance evaluated on the corresponding outer folds (Section S2). Predictive performance was assessed using the coefficient of determination *R* ^2^ and the root-mean-square error (RMSE). Uncertainty quantification and calibration were evaluated using three metrics as suggested by von Borries et al. (2026):^27^ (i) the Expected Calibration Error (ECE), (ii) the Expected Normalized Calibration Error (ENCE),^28,29^ and (iii) a distance-based calibration. The ECE and the ENCE both characterize the reliability of the predicted uncertainties. The ECE checks how closely the empirical coverage of prediction confidence intervals matches the nominal coverage (e.g., whether 95% of the predicted values actually fall within the 95% confidence interval of the predicted half-life distribution), and the ENCE evaluates whether the prediction error (residuals) scales with the predicted uncertainties. Low ECE and ENCE indicate good uncertainty calibration. The distance-based calibration evaluates whether prediction uncertainties reflect the test compounds’ distance to the training data domain. To this end, we computed the average Tanimoto similarity to the five nearest training compounds for each test molecule based on Morgan fingerprints. The correlation between distance and prediction uncertainties was assessed using Pearson’s *r* and Spearman’s ρ correlation coefficients (Section S3 for more details).

A final GPR model with optimized hyperparameters and descriptors from the best performing combination (Table S4) was trained on the complete data set and used for all subsequent DT_50,pred_ predictions.

### Half-Life Prediction for Pesticide TPs

To evaluate the persistence of pesticide TPs lacking experimental data, we extracted all TPs for which no experimental biotransformation half-life had been reported in the corresponding OECD 307 studies from the EAWAG-SOIL data package. For each TP, the final GPR model was used to predict log DT_50,pred_ and associated model uncertainty log DT_50,std_. We then calculated the probabilities *p*(P) and p­(vP) (as defined in eq 1).

### Half-Life Prediction for ZeroPM Substances

To assess the chemical coverage of our model with regard to the space of marketed chemicals, we predicted soil half-lives for all substances in the ZeroPM database (downloaded from PubChem on 10 October 2025, https://pubchem.ncbi.nlm.nih.gov/source/25168).^30^ logDT_50,mean_ and logDT_50,std_ were predicted for all compounds using the final GPR model. The predictions were visualized on a tSNE representation of the ZeroPM chemical space using COMPASS^31^ (https://github.com/BoCHemia/COMPASS).

### Code & Data Availability

Data, code, and documentation required to reproduce all results presented here can be found in the PEPPER GitHub repository (https://github.com/FennerLabs/pepper, v.1.2.0). We use streamlit to provide an intuitive user interface for predicting half-lives for single chemicals or batches of substances. The PEPPER app is freely available at https://pepper-app.streamlit.app/.

## Results and Discussion

### Data Uncertainty and Regulatory Thresholds

Running multiple soil biodegradation simulation studies for regulatory purposes typically results in a distribution of experimentally observed biotransformation half-lives for a single compound. In addition, the number of reported half-lives per compound may vary significantly: Compounds with strong data support show low uncertainty, whereas those with fewer or more variable measurements exhibit higher uncertainty. In regulatory practice, however, a substance is considered persistent if its transformation half-life in soil exceeds 120 days and very persistent if it exceeds 180 days.^2^ But how can we apply a single-value thresholds to a uncertain distribution of half-lives?

To capture data variability and uncertainty, we obtained Bayesian estimates of the mean log half-life (μ_mean_), its associated uncertainty μ_std_, and the experimental variability (σ_mean_) for the 306 pesticides and 561 TPs (867 compounds in total) in EAWAG-SOIL with reported half-lives. The number of reported half-lives *n* per compound ranges from 1 to 59, resulting in a wide range of uncertainties (Figures S2 and S3). For compounds with a single reported half-life (*n* = 1), the estimated μ_std_ depends on the chosen prior. With a justified prior, this dependence is not a limitation but a useful feature of Bayesian inference, enabling sound characterization of half-life distributions when experimental evidence is sparse. Although the influence of the prior parameters on calculated P/vP probabilities is minimal (Section 1.3—Figures S5–S9), prior assumptions should be kept in mind when interpreting the Bayesian-inferred experimental variability and associated uncertainty for compounds with few reported half-lives. The aim of this study was to predict mean half-lives. We therefore focused on the mean half-life μ_mean_ and its uncertainty μ_std_. Analysis and modeling of the experimental variability σ_mean_ is beyond the scope of this study, yet may be investigated in the future.

To understand the implications of the uncertainty relative to the fixed regulatory thresholds, we classified the compounds into nP, P or vP categories based on (i) their mean estimates μ_mean_, and (ii) a conservative scenario defined by the upper bound of the 95% CI determined by μ_std_ uncertainty around μ_mean_ (Figure 2). When the mean estimates alone determine the classification, every compound falls neatly into one category (Figure 2, left). However, once uncertainty is considered (Figure 2, right), the classification becomes ambiguous because the CI may span several categories. This ambiguity is particularly evident for compounds supported by only a single experimental half-life (*n* = 1). Examples of those include the compound 8-hydroxyquinoline (μ_mean_ = 0.19, μ_std_ = 0.97, *n* = 1), which has a probability of 3% of being classified as P, and the TP SYN505866 (μ_mean_ = −0.19, μ_std_ = 1.5, *n* = 1) of the parent compound pymetrozine, which exhibits a 5% chance of being classified as vP. These probabilities reflect the well-founded prior assumptions about the distribution of experimentally measured half-lives, underscoring a key limitation: a single half-life is often insufficient to confidently assign a compound as being nonpersistent. Consequently, it is essential to explicitly account for the half-life uncertainty in our modeling approach.^32^

**Figure 2:**
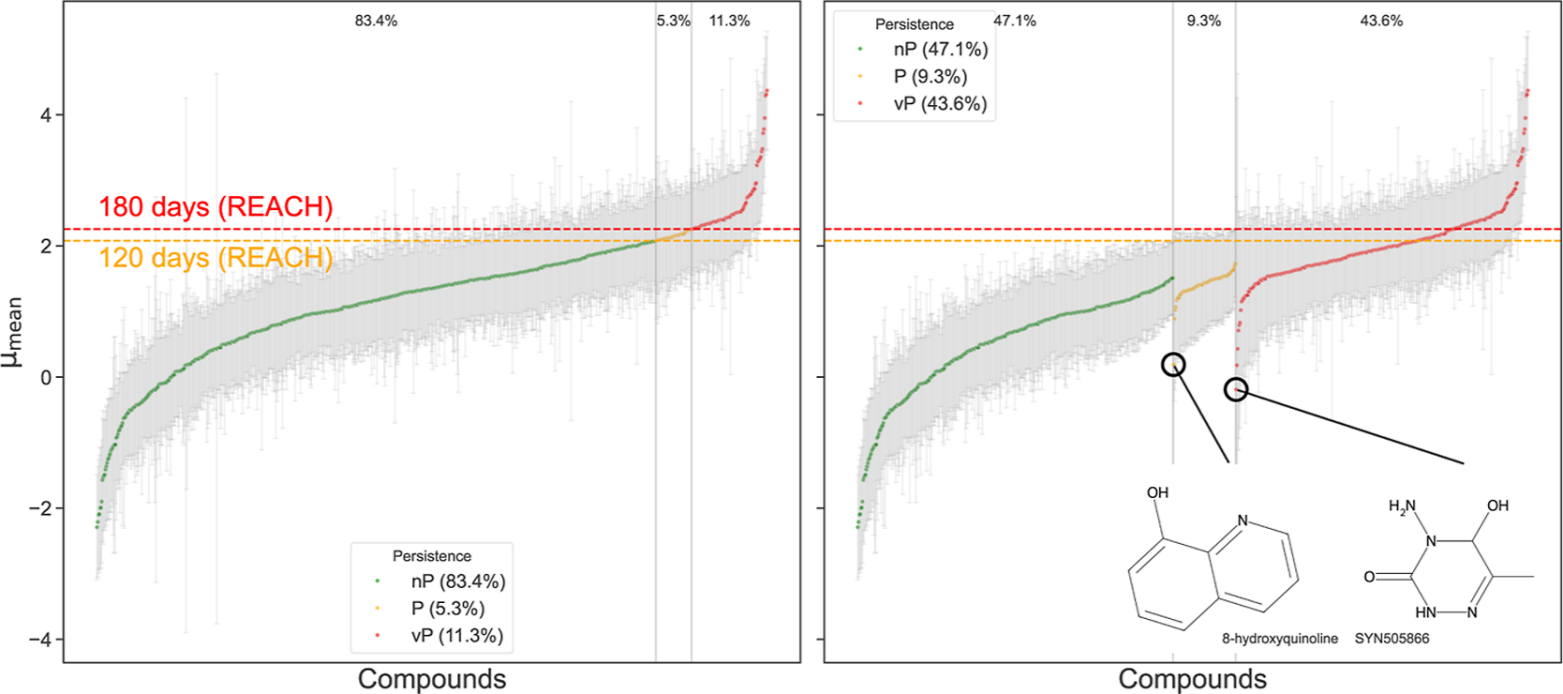
REACH persistence classification. Each marker shows the μ_mean_ for a single compound, with error bars indicating 95% CI of the Bayesian-inferred distribution. Nonpersistent (nP), persistent (P) and very-persistent (vP) compounds are colored green, yellow and red, respectively. Dashed lines denote the DT_50_ thresholds corresponding to 120 days (P criterion, yellow) and 180 days (vP criterion, red). Left: classification into REACH Persistence classes by considering μ_mean_ without the 95% CI. Right: Classification into REACH Persistence classes by considering μ_mean_ with the upper limit of the 95% CI. The percentages of the REACH persistence classes are denoted in the Figure. Molecular structures for two extreme case TPs are shown: 8-hydroxyquinoline (μ_mean_ = 0.19; μ_std_ = 0.97; *n* = 1; *p*(nP, P, vP) = 97, 3, 2%) and SYN505866 (μ_mean_ = −0.19; μ_std_ = 1.5; *n* = 1; *p*(nP, P, vP) = 93, 7, 5%).

### Building Half-Life Models with Prediction Confidence

In 5-fold cross-validation across all model—feature—feature reduction combinations, predictive performance was moderate with *R* ^2^ ranging from 0.11 to 0.32 and RMSE from 0.79 to 0.90. PaDEL descriptors consistently produced top results (Figure 3, top panel). For GPR, PaDEL descriptors yielded the highest overall performance with *R* ^2^ = 0.32, RMSE = 0.79, while for RF the best model was achieved when using all features *R* ^2^ = 0.28, RMSE = 0.81. Importantly, feature reduction with PCA did not improve the performance of the GPR over all feature—feature reduction pairs (*R* ^2^ = 0.32 with PCA), indicating that the full descriptors set contains information relevant for prediction. Both GPR and RF models reproduce the central region of the target value distribution reasonably well, but systematically overpredict short half-lives and under-predict very long ones (Figure 3, middle row). While the models capture a broad trend in soil μ_mean_, their quantitative accuracy remains limited, with a tendency to predict the mean (around 1.2 log­(days)). In addition to the predicted mean half-life DT_50,pred_, we get a model uncertainty estimate DT_50,std_ by GPR. The uncertainty estimates for the best GPR model range from 0.21 to 0.91 log­(days) (Figure 3, bottom row). RF, in contrast, produces uncertainty estimates from the tree ensemble variance that span a broader range from 0.26 to 1.58 log­(days). The more irregular appearance of the RF uncertainty histogram arises from the discrete nature of ensemble-based variance estimates, whereas GPR yields continuous uncertainty values that lead to smoother empirical distributions. An in-depth analysis showed that predictive uncertainty increases with the chemical distance from the training set (Section S4). This behavior aligns with a fundamental assumption of QSAR-modeling, namely that similar structures have similar activity. When predictions move farther from the chemical space represented in the training data, uncertainty should increase accordingly, signaling reduced reliability. Additionally, both models provide well calibrated uncertainties (Figures S10 and S11), with GPR showing slightly better values (ECE: GPR = 1.1%, RF = 4.8%; ENCE: GPR = 20.5%, RF = 15.8%). In particular, the low ENCE value indicates that the model’s uncertainties correspond well to its residuals,^28^ or in other words, that small uncertainties are associated with small errors, and large uncertainties with larger deviations. Overall, we find GPR uncertainties to be superior over the RF uncertainties for the following reasons: First, the training compound uncertainty is incorporated during training, respecting the fact that already the training data is highly uncertain (Figure S12). Second, to calculate the probability of exceeding the REACH thresholds (eq 1) we require normally distributed model uncertainty. This is an inherent feature for GPR, but not necessarily true for the empirical standard deviation of the RF tree spread. For these reasons, GPR was selected as the final model (see Table S4).

**Figure 3:**
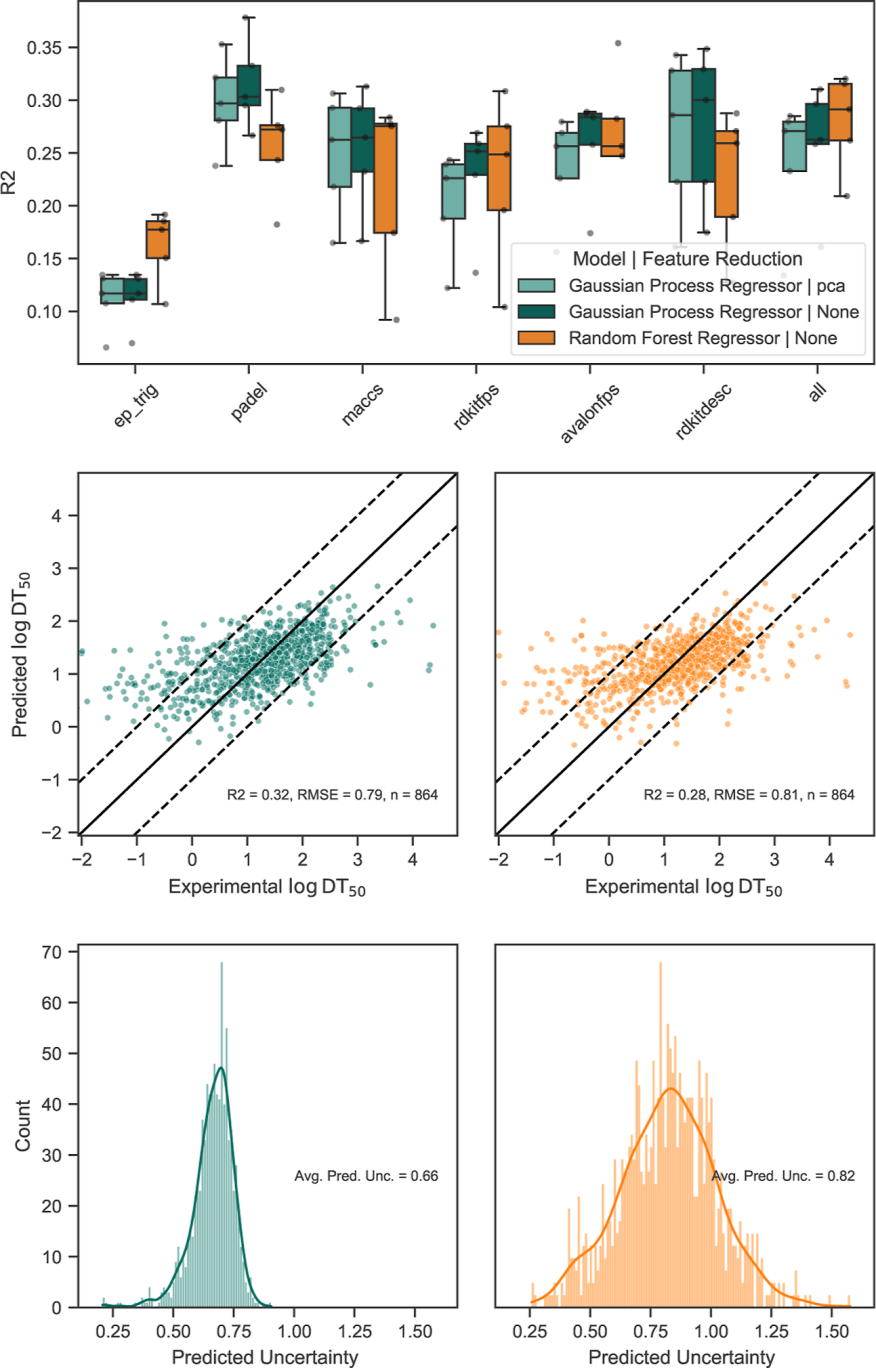
Model performances, prediction accuracy and uncertainty estimates for GPR and RF models (Top) *R* ^2^ results from 5-fold nested cross-validation of different descriptor and feature reduction combinations. (Middle) Parity plots of test compounds for the best GPR (left) and RF (right) models using PaDEL descriptors. (Bottom) Distributions of predictive uncertainties for GPR (left) and RF (right). Avg. Pred. Unc.: Average prediction uncertainty.

### Applicability Domain and Limitations

Several aspects should be considered when interpreting model predictions. The applicability domain (AD) includes single molecular structures (excluding composite mixtures), small molecules (molecular weight <1200 Da), and only organic molecules. Even if a molecule falls within the AD, the prediction reliability depends on how well the compound is represented in the training chemical space. For poorly represented compound classes, the model tends to shrink predictions toward the mean of the training distribution, but this effect is less pronounced for predictions with lower uncertainty (Figure S10). Importantly, while point estimates become less informative in these cases, the calibrated uncertainties are trustworthy and provide meaningful guidance in prediction interpretation. As a guidance, we suggest 0.7 as an upper threshold for an “acceptable” prediction confidence, which corresponds to a 5-fold change in half-live [days] and to the average prediction uncertainty in the test sets, and 0.5 as a threshold for a “good” prediction, which approximately corresponds to a 3-fold change. Given the good performance of the prediction uncertainty to evaluate the accuracy of a prediction, we did not consider providing an additional similarity-based AD metric, which would be more difficult to interpret and lack statistical calibration. The following applications show use cases with respect to the model’s applicability domain. Predictions for pesticide TPs (Application 1) correspond to interpolation within the chemical space covered by the training data, whereas predictions for marketed chemicals (Application 2) correspond to extrapolation outside the training space.

### Application 1: Predicting Half-Lives for Pesticide TPs without Kinetic Data

Biotransformation pathways compiled in the EAWAG-SOIL package^19^ represent experimentally derived networks that describe how a parent compound is biotransformed into its TPs. Experimentally, such pathways are determined through studies in which a radio-labeled test parent compound is introduced into a controlled soil environment. The disappearance of the parent compound over time (concentration–time series) is used to determine the transformation half-life of the parent, while radio-label tracking combined with chromatographic and spectrometric techniques identifies and quantifies TPs. According to OECD guidelines,^33^ TPs are classified based on their abundance relative to the applied parent compound. Major TPs (≥10% of the applied radioactivity at any time during the study) must undergo further evaluation in most cases, while minor TPs (<10% of applied radioactivity) represent detectable but less abundant metabolites in the study, which are not automatically subjected to separate persistence assessment. This poses a certain problem: low abundant TPs may remain uncharacterized despite their potential environmental relevance.^34^ We wanted to know whether any of the EAWAG-SOIL TPs without kinetic data might be persistent, independent of them being labeled as “major” TPs or not. Therefore, we extracted all TPs without half-lives from every parent compound pathway in the soil data package, resulting in 819 unique TPs from 245 pathways.

The predicted mean log DT_50,pred_ values for the 819 TPs without kinetic information spanned a range from −0.11 to 2.47 log days. The associated predicted standard deviations were 0.39 to 1.12 log days with a mean of 0.68 log days. To focus on predictions with acceptable model uncertainty, we filtered out predictions with a log DT_50,std_ ≥ 0.7 log days, which resulted in 493 TPs (i.e., 60% of all TPs without kinetic information), for which the GPR expressed acceptable to good confidence (Table S5). Comparison across pathways showed that TPs are predicted to be either less or more persistent than their corresponding parent compounds (SI Figure S14). Only a minority of TPs exhibited predicted means near or above the REACH thresholds, suggesting that some low-abundance TPs may still call for further investigation despite not being reported as major products. Unsurprisingly, these are mostly TPs that are formed from persistent (pyridaben, fenbuconazole) and very persistent pesticides (ipconazole, tebuconazole, epoxiconazole, bromuconazole, oxadiazon, tetraconazole). One exception is a TP of metazochlor (BH479–7, μ_mean_ = 1.19) with a predicted mean half-life of 2.07 log days (log DT_50,std_ = 0.64) and a *p*(P) of 49%. All derived persistence class probabilities and predictions are provided in the Supporting Information “Predictions_pesticide_TPs.csv”.

Selected pathway examples in Table 1 illustrate how the model differentiates persistence among parent compounds and their TPs. For the picoxystrobin pathway (Figure S15), the model predicts the TP IN-QDY63 to have a notably longer half-life and a higher probability of being P than both the parent compound and the major TP IN-QDK50 (Table 1). The pathway of the very persistent substance bromuconazole (Figure S16) produces a TP (MWt 257) predicted to be clearly nonpersistent and one TPs (LS802050) predicted to show low *p*(P), while five TPs (LS860976, LS 860551, LS861590 and RPA 401527, LS-870353) show increased probabilities of being P or vP (Table 1). Although most TPs (88%) lack major/minor classification in EAWAG-SOIL (SI Table S7), we can observe a general trend that minor TPs have shorter half-lives than major TPs across reported and predicted half-lives (Figure S17). However, a “minor” classification does not guarantee fast degradation, as can be seen for the minor TPs TPSA (SSRE-001) (μ_mean_ = 2.9, μ_std_ = 0.21, *p*(P) = 100%) of the flazasulfuron pathway and RP 36221 (μ_mean_ = 2.88, μ_std_ = 0.14, *p*(P) = 100%) of the iprodine pathway (Figure S18).

**Table 1:** Comparison of Selected Parent Compounds and Transformation Products (TPs) With Half-Lives and Persistence Class Probabilities^a^

parent	compound	log DT_50,mean_	log DT_50,std_	*p*(nP) (%)	*p*(P) (%)	*p*(vP) (%)
Picoxystrobin	parent	1.37	0.06	100	0	0
	IN-QDK50	1.19	0.14	100	0	0
	IN-QDY63*	1.54	0.52	85	15	8
Bromuconazole	parent	2.75	0.14	0	100	100
	MWt 257*	0.62	0.45	100	0	0
	LS860976*	2.47	0.58	25	75	65
	LS 860551*	1.67	0.60	75	25	17
	LS861590*	1.57	0.64	79	21	14
	RPA 401527*	2.07	0.66	50	50	39
	LS802050*	1.05	0.65	94	6	3
	LS-870353*	1.56	0.63	80	20	13

a Pathway links to enviPath: Picoxystrobin, Bromuconazole. IN-QDK50 is a major TP. *Values for these compounds are predicted.

Together, these examples show that even under moderate predictive performance, the model can reveal TPs that are likely to warrant further examination. These pathway level evaluations illustrate how computational screening can be used to highlight low-abundance TPs that might be of regulatory interest.

### Application 2: Predicting Half-Lives for Marketed Chemicals

As most marketed chemicals lack half-life data,^14^ we wanted to know for how many of them we could predict half-lives with confidence. To do this, we applied the final GPR model on all substances collected in the ZeroPM data set of marketed chemicals.^30^ Out of the initial 137,915 substances, 95,783 were within the model’s AD (salts: 41,105, >1200 Da: 1682), and PaDEL descriptors could be calculated for 94,834 of them. For 353 ZeroPM substances we could find experimental half-lives in the model’s training data, which is slightly more than the number of pathways (and therefore parent compounds) in the EAWAG-SOIL data set (317). When examining the distribution of predicted half-lives and uncertainties, we observe that most substances with high prediction confidence are present in the training data, whereas the bulk of the prediction uncertainties lies between 0.6 and 1.2 standard deviations (Figure S19). We could further identify 26 ZeroPM substances with very high (>80%, 12 substances) or high (70–80%, 14 substances) probabilities of being persistent. In-depth analysis showed that 9 of the 12 substances with very high *p*(P) were stereoisomers or isotopomers of a persistent training compound and therefore not further analyzed. For the remaining three substances with very high *p*(P), no information on environmental persistence could be found in scientific literature and available regulatory reports. Six substances assigned high *p*(P) were further analyzed (Figure S20): Fluconazole and nalidixic acid have been show to have low removal rates in WWTP.^35,36^ No information on persistence could be found for the quinolone antibiotics rosoxacin and piromidic acid, as well as for voriconazole, a triazole antifungal that is structurally similar to fluconazole, and for the pesticide etaconazole, which is structurally very similar to the persistent propiconazole (predicted *p*(P) of 68%, classified as persistent according to EFSA^37^). These examples provide a qualitative confirmation that the predictions of our model are reasonable and useful to screen for potentially persistent chemicals.

Recent work by von Borries et al. has estimated that currently available soil half-life data, representing 1.3% of marketed chemicals, can be used reasonably to extrapolate to 8% of marketed chemicals.^14^ When looking at the top 8% of substances with highest confidence, their log DT_50,std_s lie below 0.7 (max = 0.67, mean = 0.63, *n* = 11033), which corresponds to our upper threshold for an “acceptable” uncertainty.

When visualizing the ZeroPM predictions on a 2-dimensional t-SNE projection of the marketed chemicals space using COMPASS (https://github.com/BoCHemia/COMPASS),^14,31^ we could observe the following patterns (Figure 4): In general, predicted half-lives were longer on the bottom right side of the map where benzenoids and organoheterocyclic compounds were most prevalent. In particular, the cluster of per- and polyfluoroalkyl substances (PFAS) (around TSNE1 = 120, TSNE2 = 50) is a hot spot of both high uncertainty and high persistence probabilities, as well as the larger area on the bottom right (around TSNE1 = (100 to 130), TSNE2 = (−100 to −160)), where large organoheterocyclic compounds are present, many of them reported as mixtures and therefore beyond the model’s AD. A smaller uncertainty hot spot can be found in the center part of the map (around TSNE1 = −50, TSNE2 = −60), where large, natural product-like structures such as peptides and hormones are clustered. Unlike PFAS, despite the high log DT_50,std_, these compounds are not flagged as *p*(P). Another uncertainty cluster could be identified for brominated compounds (TSNE1 = 15, TSNE2 = −100). Increased *p*(P) was observed for benzenoids with multiple aromatic rings (TSNE1 = 50, TSNE2 = 120) and for sulfonic acids (TSNE1 = 130. TSNE2 = 0). In the upper left part of the map, shorter half-lives with moderate uncertainty seem to dominate, although the training data is sparse in this area. A closer look reveals that this space is mostly populated by hydrocarbons, lipids and lipid-like structures, organic oxygen compounds, and organic acids and derivatives - so rather low-molecular weight substances that are structurally similar to naturally occurring compounds. The mixed metal/non metal compounds were beyond the AD and are visible as a gray cluster in the center of the map (around TSNE1 = 70, TSNE2 = 40). These examples illustrate the capability of the model to highlight potentially persistent substances for further investigation, and to identify knowledge gaps to direct future data collection efforts.

**Figure 4:**
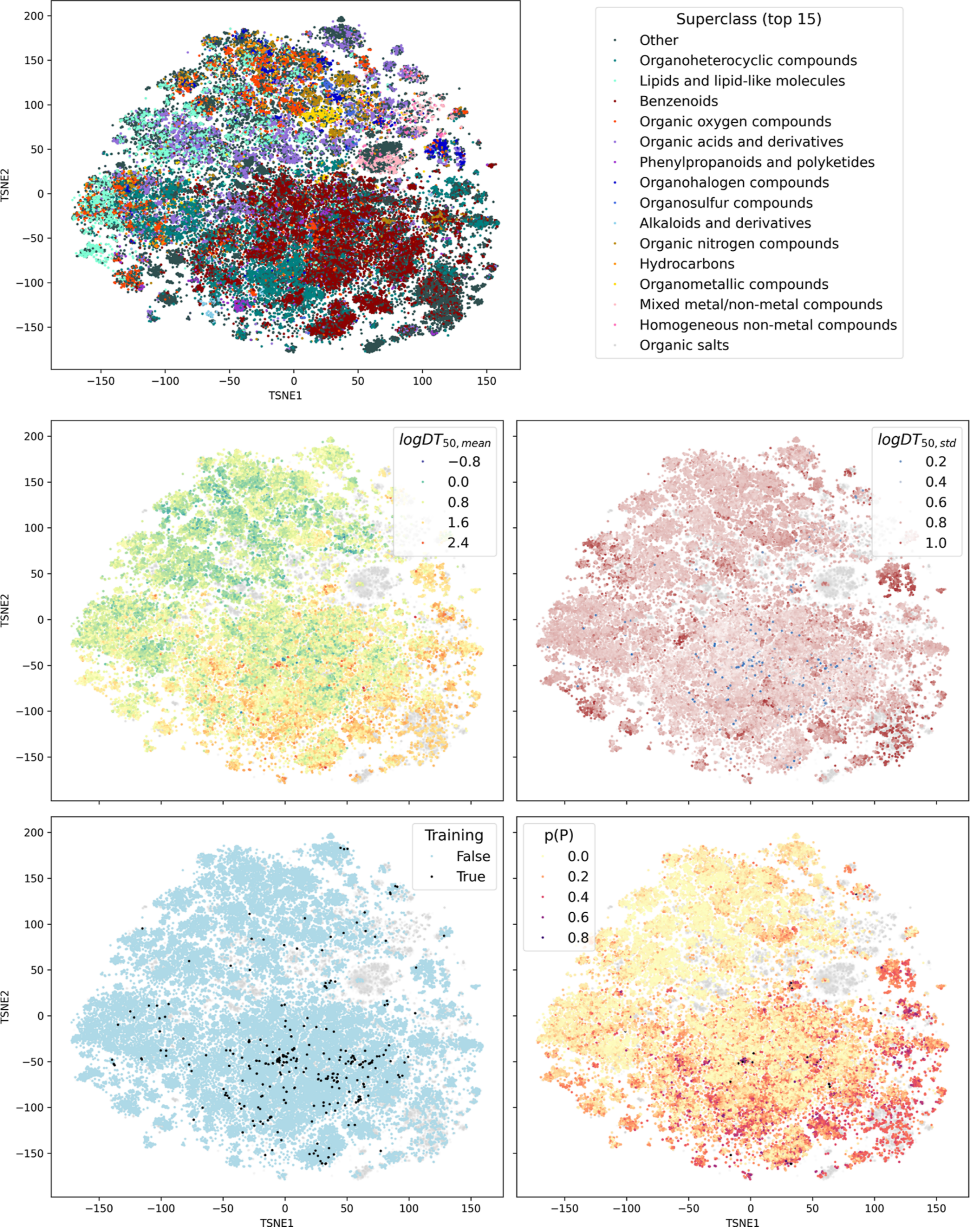
Distribution of prediction uncertainty and persistence potential within the space of marketed chemicals. Top: 2-dimensional representation of the ZeroPM substances, colored by chemical classification. Middle: Predicted mean half-lives (left) and model uncertainties as standard deviations (right) for ZeroPM chemicals. Bottom: Presence in training data (left) and probability of being persistent (right) for ZeroPM chemicals. Gray dots indicate compounds in the ZeroPM data set that were outside the applicability domain of our model. The chemical space plots were created using COMPASS.^31^

### Implications and Outlook

Despite showing low average performance metrics, the soil half-life model presented here proved useful to screen long lists of chemicals for high persistence and knowledge gaps. In general, we expected higher model confidence for chemicals that are similar to the training data (i.e., pesticides). Indeed, the average uncertainty for ZeroPM compounds is 0.75 log days (model extrapolation), which is slightly higher than the 0.68 log days for pesticide TPs (model interpolation), which are structurally more similar to the training compounds. Importantly, when benchmarking our model against two popular tools for half-life prediction in soil on an external test set of 25 compound (12 pharmaceuticals, 7 industrial chemicals, and 6 pesticides), our model (*R* ^2^ = 0.28, RMSE = 1.46) outperformed both EPISuite Biowin4 ^12^ (*R* ^2^ = 0.18, RMSE = 1.51) and VEGA^13^ (*R* ^2^ = 0.05, RMSE = 1.62) (Section 4.2, “Validation on external data set”).

We would like to emphasize that the experimental variability was not modeled per se, although it has an influence on the uncertainty of the mean. In the future, additionally modeling experimental variability would make it possible to predict a distribution of possible experimental outcomes for a given substance. Such a model could also be designed to correct for environmental and experimental parameters, enabling the prediction of a half-life under specified conditions. Including such parameters in a model setting has not been attempted here, as previous work has shown that finding influencing factors in the soil data set is not trivial due to nonlinear relationships and a sparsely populated parameter matrix.^19^

Another aspect not discussed in this study is the interpretability of the model because identifying molecular descriptors with high influence is not straightforward for GPR. Yet, we believe that future efforts should be directed at improving the design and/or selection of molecular descriptors and fingerprints with interpretable roles in persistence prediction.

We also plan to extend our modeling approach to other end points, such as half-lives observed in water-sediment systems according to OECD guideline 308 ^33^ or to biotransformation rate constants observed in activated sludge. Corresponding data sets are available in enviPath, as EAWAG-SEDIMENT and EAWAG-SLUDGE (https://envipath.org/package). In parallel, continued data collection efforts are crucial to improve the coverage of chemical space and the confidence in the reported end points. Given the under-representation of extreme half-life values in our data set, future data collection efforts should further prioritize the integration of censored values into training data sets.

Our approach can enhance prediction transparency, provide a more realistic understanding of model confidence, and ultimately support regulatory decision-making under uncertainty. We hope that this work will help regulation in prioritizing potentially problematic substances for experimental testing and contribute to developing chemicals that are degradable by design.^16,38^

## Supplementary Material







## Data Availability

The models are integrated into the PEPPER framework,^22^ which is available on GitHub (https://github.com/FennerLabs/pepper) and as a Python library on PyPi (pepper-lab) to facilitate model integration into downstream applications. We also provide a graphical user interface at https://pepper-app.streamlit.app/, where users can enter single or lists of SMILES (batch mode) and obtain predictions in seconds.

## Notes

Predictions_pesticide_TPs: Predicted half-lives and uncertainties for all TPs in EAWAG-SOIL lacking kinetic data (CSV)
Predictions_ZeroPM: Predicted half-lives and uncertainties for all ZeroPM substances within the applicability domain of the model (CSV)
Detailed description of methods and additional results, supporting figures and tables (PDF)

## Abbreviations

P: Persistence/persistent
vP: very persistent
nP: nonpersistent
ECHA: European Chemical Agency
SSbD: Safe and Sustainable by Design
QSAR: Quantitative Structure–Activity Relationships
RB: Ready biodegradability
OECD: Organization for Economic Co-operation and Development
BI: Bayesian Inference
RF: Random Forest
GPR: Gaussian Process Regression
REACH: Registration, Evaluation, Authorisation and Restriction of Chemicals
WWTP: Wastewater Treatment Plant
BT-rules: Biotransformation rules (enviPath)
PCA: Principal Component Analysis
RBF: Radial Basis Function
RMSE: Root-mean-square error
ECE: Expected Calibration Error
ENCE: Expected Normalized Calibration Error
TP: Transformation Product
CI: Credible Interval
