# Supplementary material for: Confidently Uncertain: Probabilistic Machine Learning to Predict Soil Biotransformation Half-Lives
Source: Environ Sci Technol. 2026 Apr 2;60(14):11077–86. doi: 10.1021/acs.est.6c03516 (PMC13085514; doi:10.1021/acs.est.6c03516)
Supplement: Supplementary file 3 [file es6c03516_si_003.pdf]

Supporting Information: Confidently uncertain: Probabilistic machine learning to predict soil biotransformation half-lives

Moritz Salz,[†] José Andrés Cordero Solano,[†] Kathrin Fenner,[*,†,‡] and Jasmin Hafner[†,‡]

†*Department of Environmental Chemistry, Eawag, Dübendorf, Switzerland*

‡*Department of Chemistry, University of Zurich, Zürich, Switzerland*

E-mail: kathrin.fenner@eawag.ch

List of Figures

S1 **Soil experimental value distribution.** The distributions of the descriptive mean and the standard deviation of the log-transformed half-lives are shown as a kernel density estimation (KDE) for of all the compounds (blue) and for a reference data set of only compounds with 20 or more reported half-lives (green). The red area shows the distribution of the Bayesian-inferred log half-lives. The descriptive means of the reference chemicals are distributed with a mean of 1.22 log(days) and a standard deviation of 0.67 log(days), and the descriptive standard deviations representing experimental variability are distributed with a mean of 0.38 log(days) and a standard deviation of 0.12 log(days). S-11

S2 Distribution of Bayesian inferred log half-life means (μ_{mean}) and mean uncertainties (μ_{std}) . mean: mean value of the distribution; std: standard deviation of the distribution. S-11

S3 Dependence of uncertainty of μ_{mean} on the number of experimental values per compound. S-12

S4 **Predicted persistence probability distribution for an example compound** with $\mu_{mean} = 2.4$ and $\mu_{std} = 0.24$, yielding p(nP) = 9%, p(P) = 91%, and p(vP) = 73%. The solid black curve shows the Gaussian predictive distribution $\mathcal{N}(\mu_{\text{mean}}, \mu^2_{\text{std}})$. Vertical dashed lines mark the log(120) and log(180) thresholds for persistent (P) and very-persistent (vP), respectively. Shaded areas denote p(nP) (blue), p(P) (orange), and p(vP) (red hatched) S-13

S5 Varying prior parameter μ_{mean}. Each subplot shows the estimated half-life distribution (posterior mean μ_{mean} and its uncertainty μ_{std}) for a selected compound with n reported half-lives. Blue: μ_{mean}=0.5, black: μ_{mean}=1, red: μ_{mean}=1.5. The dashed and the dotted lines indicate the threshold values for P (120 days) and vP (180 d), respectively. S-14

S6 Varying prior parameter μ_{std}. Each subplot shows the estimated half-life distribution (posterior mean μ_{mean} and its uncertainty μ_{std}) for a selected compound with n reported half-lives. Blue: μ_{std}=1.5, black: μ_{std}=2, red: μ_{std}=2.5. The dashed and the dotted lines indicate the threshold values for P (120 days) and vP (180 d), respectively. S-15

S7 Varying prior parameter σ_{mean}. Each subplot shows the estimated half-life distribution (posterior mean μ_{mean} and its uncertainty μ_{std}) for a selected compound with n reported half-lives. Blue: σ_{mean}=0.3, black: σ_{mean}=0.4, red: σ_{mean}=0.5. The dashed and the dotted lines indicate the threshold values for P (120 days) and vP (180 d), respectively. S-16

S8 Varying prior parameter σ_{std}. Each subplot shows the estimated half-life distribution (posterior mean μ_{mean} and its uncertainty μ_{std}) for a selected compound with n reported half-lives. Blue: σ_{std}=0.3, black: σ_{std}=0.4, red: σ_{std}=0.5. The dashed and the dotted lines indicate the threshold values for P (120 days) and vP (180 d), respectively. S-17

S9 Varying prior parameter σ_{min}. Each subplot shows the estimated half-life distribution (posterior mean μ_{mean} and its uncertainty μ_{std}) for a selected compound with n reported half-lives. Blue: σ_{min}=0.1, black: σ_{min}=0.2, red: σ_{min}=0.3. The dashed and the dotted lines indicate the threshold values for P (120 days) and vP (180 d), respectively. S-18

S10 **Performance and uncertainty analysis of the Gaussian Process Regression (GPR) model trained on PaDEL descriptors using five-fold cross-validation.** Top left: parity plot of predicted versus experimental soil half-lives (log DT_{50}). Top right: distribution of predictive uncertainties ($DT_{50,std}$) across folds. Middle left: confidence calibration plot comparing nominal and empirical coverage. Middle right: relationship between prediction error and predicted uncertainty. Bottom left: dependence of predictive uncertainty on chemical distance to the training set. Bottom right: parity plot of predictions with a prediction score below 0.5. Results are aggregated over all outer cross-validation folds. S-27

S11 **Performance and uncertainty analysis of the Random Forest (RF) model trained on PaDEL descriptors using five-fold cross-validation.** Top left: parity plot of predicted versus experimental soil half-lives (log DT_{50}). Top right: distribution of predictive uncertainties derived from ensemble variance across folds. Middle left: confidence calibration plot comparing nominal and empirical coverage. Middle right: relationship between prediction error and predicted uncertainty. Bottom left: dependence of predictive uncertainty on chemical distance to the training set. Bottom right: parity plot of predictions with a prediction score below 0.5. Results are aggregated over all outer cross-validation folds. S-28

S12 **Training set fitted uncertainty (predicted) vs μ_{std} (experimental std)** for RF (left) and GPR (right). S-29

S13 Comparison of PEPPER, BIOWIN4, and VEGA prediction performance on the external data set. Error bars represent the 95% confidence intervals for the logDT50 uncertainty of the mean on the y-axis, and the prediction uncertainty for PEPPER predictions on the x-axis. S-31

S14 The Bayesian estimated μ_{mean} values for parent compounds (red points) and their associated transformation products with predicted values $\mathrm{DT}_{50,\mathrm{pred}}$ (blue cross) filtered to $\mathrm{DT}_{50,\mathrm{std}} \leq 0.7$ log days. The horizontal bars are the 95 % intervals of the parent compounds (μ_{std}) and TPs ($\mathrm{DT}_{50,\mathrm{std}}$). Vertical dashed lines indicate the REACH persistence thresholds for persistent (P) and very persistent (vP) classification. S-32

S15 Picoxystrobin pathway in enviPath with reported (black box) and predicted (red boxes) half-lives, prediction uncertainties ($\log \mathrm{DT}_{50,\mathrm{std}}$), and persistence probabilities (p(P)). The red circle indicates the parent compound. $\log \mathrm{DT}_{50,\mathrm{pred}}$: predicted log half-life; $\log \mathrm{DT}_{50,\mathrm{std}}$: prediction log half-life uncertainty; μ_{mean}: Bayesian inferred mean log half-life; μ_{mean}: Bayesian inferred mean log half-life uncertainty S-33

S16 Bromuconazole pathway in enviPath with reported (black box) and predicted (red boxes) half-lives, prediction uncertainties ($\log \mathrm{DT}_{50,\mathrm{std}}$), and persistence probabilities (p(P)). The red circle indicates the parent compound. $\log \mathrm{DT}_{50,\mathrm{pred}}$: predicted log half-life; $\log \mathrm{DT}_{50,\mathrm{std}}$: prediction log half-life uncertainty; μ_{mean}: Bayesian inferred mean log half-life; μ_{mean}: Bayesian inferred mean log half-life uncertainty S-34

S17 **Distribution of reported (dots) and predicted (crosses) biotransformation half-lives for different TP importance classifications.** The prediction confidence is indicated as $\log \mathrm{DT}_{50,\mathrm{std}}$ on a blue-red color scale. TPs classified as "mixed" are assigned "minor" and "major" in different experiments or pathways. TPs classified as "nan" have no TP importance assigned. The boxplots are derived from the sum of reported and predicted $\log \mathrm{DT}_{50}$. . S-35

S18 **Examples of minor TPs with high reported half-lives.** (top) TPSA (SSRE-001), a minor TP of the flazasulfuron pathway. (bottom) RP 36221, a minor TP of the iprodione pathway. The Bayesian inferred mean estimates (μ_{mean}), associated uncertainty (μ_{std}) and probability of persistence (p(P)) are reported, illustrating that a TP classified as minor does not necessarily imply fast degradation. S-36

S19 **Predicted mean $\log \mathrm{DT}_{50,\text{pred}}$ and associated uncertainty $\log \mathrm{DT}_{50,\text{std}}$ for 95,013 marketed chemicals in the ZeroPM database.** The colors indicate the predicted probability of each substance to be persistent (P), and crosses mark chemicals with experimental half-lives in the training data. The vertical dotted and dashed lines indicate the P and vP thresholds at 120 and 180 days, respectively. S-37

S20 **9 out of the 26 ZeroPM substances with a predicted probability of being persistent (p(P)) higher than 70%.** Top: substances with p(P)>80%, bottom: substances with 70% > p(P) > 80%. S-38

List of Tables

S1 Prior distributions for the Bayesian half-life models. S-9

S2 Evaluated combinations of model type, molecular feature representation, and dimensionality reduction strategy. All combinations were evaluated using nested cross-validation. S-19

S3 Hyperparameter configurations evaluated for Random Forest (RF) and Gaussian Process Regression (GPR) models. All kernels were multiplied with a constant kernel with bounds (1e-3, 1e3). S-20

S4 Best-performing hyperparameter configurations for Gaussian Process Regression (GPR) and Random Forest (RF) models. GPR kernel is multiplied by ConstantKernel with bounds (1e-3, 1e3). S-21

S5 *Model Results.* Predictive performance, uncertainty calibration, and distance–uncertainty correlations for all evaluated model–feature combinations. Results are reported for Random Forest (RF) and Gaussian Process Regression (GPR) models trained on different molecular descriptor sets, with and without principal component analysis (PCA) for dimensionality reduction. Model performance is quantified using the coefficient of determination (R^2) and the root-mean-square error (RMSE). Uncertainty calibration is evaluated using the expected calibration error (ECE) and the expected normalized calibration error (ENCE). The relationship between predicted uncertainty and distance from the training domain is assessed using Spearman's and Pearson's r correlation coefficients between predicted uncertainty and the average Tanimoto distance to the five nearest training compounds. All metrics are averaged across 5-fold nested cross-validation. S-24

S6 **Comparison of PEPPER, BIOWIN4, and VEGA predictions.** logDT50 mean: logDT50 [log days] as estimated by Bayesian inference from experimental data points, logDT0 std: Uncertainty of the estimated mean as a standard deviation, PEPPER: logDT50 predicted by PEPPER, BIOWIN4: logDT50 obtained from transforming the BIOWIN4 output for primary biotransformation using the equation postulated by Arnot *et al.* (2005),[S1] VEGA: logDT0 predicted by VEGA version 1.2.6, PEPPER std: uncertainty of the logDT50 predicted by PEPPER as a standard deviation. S-30

S7 Overview on transformation products in EAWAG-SOIL with and without reported half-lives and organized by minor/major classification. S-34

S1 Data Curation & Bayesian Inference

S1.1 Bayesian Inference

Since we have multiple half-lives for most compounds, we estimated the log experimental half-life $\log DT_{50}$ distribution on a compound level using the Bayesian inference (BI) framework from Hafner *et al.* (2023).[S2] For each compound, the mean μ and standard deviation σ of the $\log DT_{50}$ distribution were inferred from the reported observations in the EAWAG-SOIL data set using the prior distributions summarized in table S1. The priors are designed to incorporate empirical knowledge about the reported means and variability present in the experimental data. The priors were derived from the distribution patterns of compounds with substantial experimental support (≥ 20 reported values per compound).[S2] This ensures that, particularly for compounds with limited experimental support, the inference is guided by realistic prior knowledge. Since we sample many of these distributions per compound, we get a our best guess of the mean half-life μ_{mean} and its uncertainty μ_{std} per compound. For simplicity, we do not use the full experimental standard deviation estimate σ_{mean} of the compounds $\log DT_{50}$ distribution in this paper.

Table S1: Prior distributions for the Bayesian half-life models.

**Parameter**	**Prior Distribution**
μ	Normal(1.5, 2)
σ	LogNormal(0.4, 0.4)
σ_{min}	0.2

Figure S1 (left) compares the distribution of posterior μ_{mean} to descriptive geometric means of compounds' experimental values. The Bayesian-inferred μ_{mean} align well with the descriptive estimates, confirming that central tendencies in the data is successfully captured with BI from Hafner *et al.* (2023).[S2] The soil dataset contains many compounds with extensive experimental support (up to 59 reported half-lives per compound). This allows the Bayesian inference model to estimate σ_{mean} based primarily on data, resulting in posterior

estimates that closely match the descriptive standard deviations (Figure S1, right). The mean half-lives of all compound range from -2.29 to 4.37 log days and are close to normally distributed with a mean of 1.23 log days (Figure S2). The mean half-life uncertainties range from 0.25 to 2.14 log days. The level of data support per compound determines the minimal value of μ_{std} that can be obtained, and the presence of censored data as well as highly variable experimental outcomes increase the estimated uncertainty (Figure S3). Compounds with many half-lives available cluster at low μ_{std}, reflecting high confidence in their inferred μ_{mean}. In contrast, compounds with only a few experimental data points or many censored data points spread toward higher μ_{std}. In other words, the more experimental data underpin an estimate, the lower the uncertainty about the inferred mean μ_{mean}. However, this trend is not without exceptions. There are compounds, such as a TP from the Pymetrozine pathway in soil, namely CGA215525, for which we have 9 reported half-lives, which range from - 2.3 to >3.0 $\log \mathrm{DT}_{50}$. This example highlights that compounds with moderate data support but wide variability can also lead to high μ_{std}. This clear dependence of uncertainty on reported half-lives highlights the importance of incorporating the standard deviation μ_{std} as heteroscedastic noise into model training, such that well-supported compounds carry more weight.

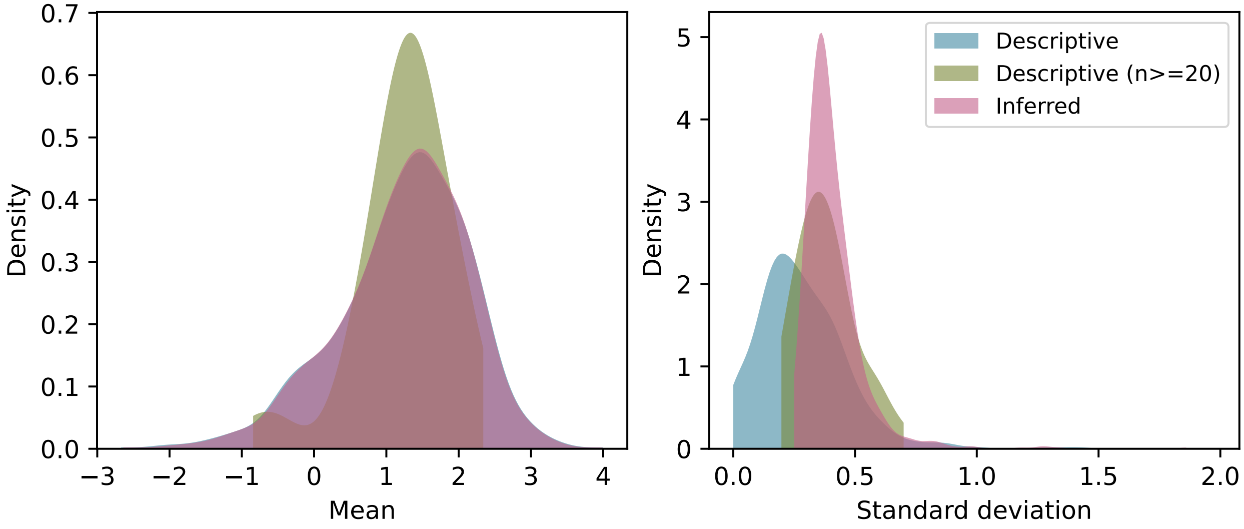

Figure S1: **Soil experimental value distribution.** The distributions of the descriptive mean and the standard deviation of the log-transformed half-lives are shown as a kernel density estimation (KDE) for of all the compounds (blue) and for a reference data set of only compounds with 20 or more reported half-lives (green). The red area shows the distribution of the Bayesian-inferred log half-lives. The descriptive means of the reference chemicals are distributed with a mean of 1.22 log(days) and a standard deviation of 0.67 log(days), and the descriptive standard deviations representing experimental variability are distributed with a mean of 0.38 log(days) and a standard deviation of 0.12 log(days).

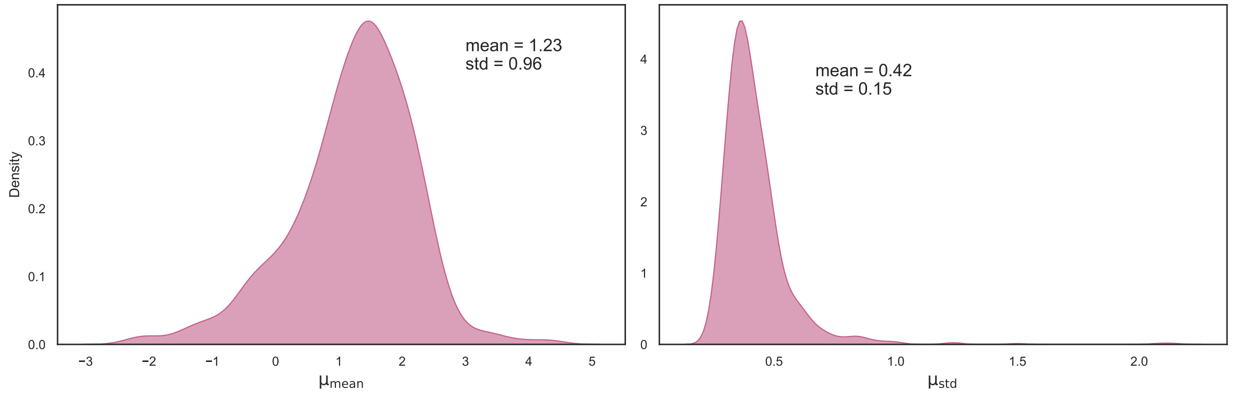

Figure S2: Distribution of Bayesian inferred log half-life means (μ_{mean}) and mean uncertainties (μ_{std}) . mean: mean value of the distribution; std: standard deviation of the distribution.

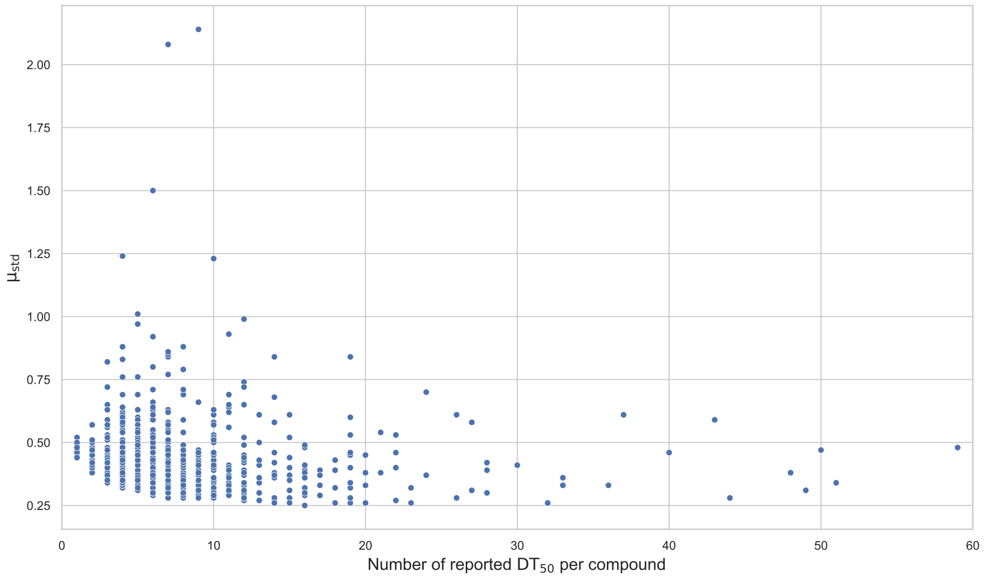

Figure S3: Dependence of uncertainty of μ_{mean} on the number of experimental values per compound.

S1.2 REACH class probabilities

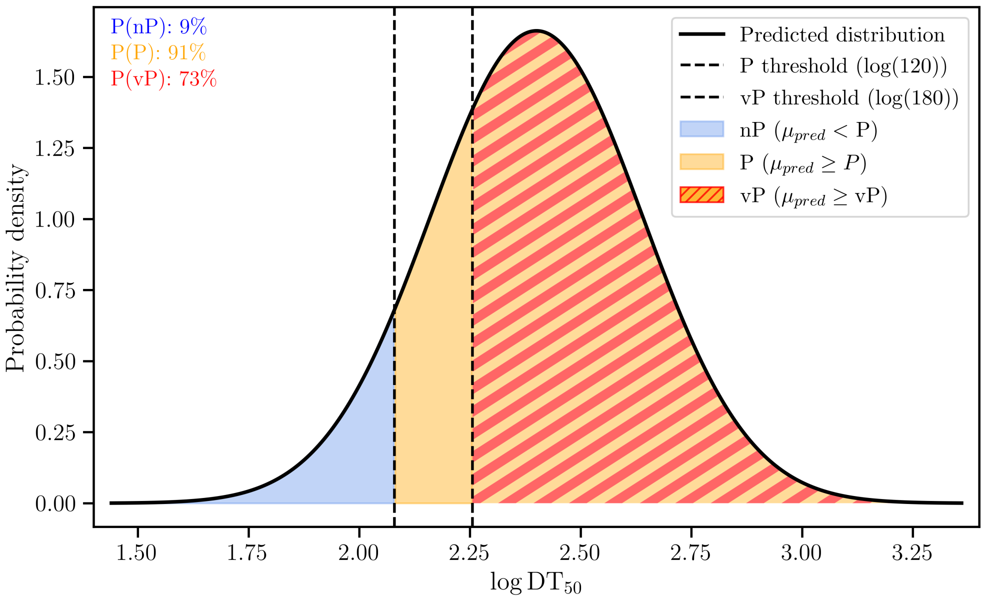

Figure S4: **Predicted persistence probability distribution for an example compound** with μ_{mean} = 2.4 and μ_{std} = 0.24, yielding p(nP) = 9%, p(P) = 91%, and p(vP) = 73%. The solid black curve shows the Gaussian predictive distribution $\mathcal{N}(\mu_{\rm mean}, \mu_{\rm std}^2)$. Vertical dashed lines mark the log(120) and log(180) thresholds for persistent (P) and very-persistent (vP), respectively. Shaded areas denote p(nP) (blue), p(P) (orange), and p(vP) (red hatched)

S1.3 Sensitivity analysis

To asses the impact of the chosen prior on the distribution of predicted persistence probabilities, we varied the five prior parameters ($\mu_{\rm mean}$, $\mu_{\rm std}$, $\sigma_{\rm mean}$, $\sigma_{\rm std}$, $\sigma_{\rm min}$) one by one and analyzed how p(P) changed as a consequence. For each prior parameter, we once increased and once decreased the original prior value (Figures S5 to S9). The analysis was performed on 9 compounds from the soil data set to represent different cases of data coverage (few vs many reported half-lives, presence of censored values). The same 9 compounds were used previously to showcase the behavior of Bayesian inference for substances of the soil dataset (Hafner et al., 2023, Figure 3).[S2]

For 6 of the 9 compounds (Tribenuron-methyl, dazomet, quizolofop-P-tefuryl, Triazine

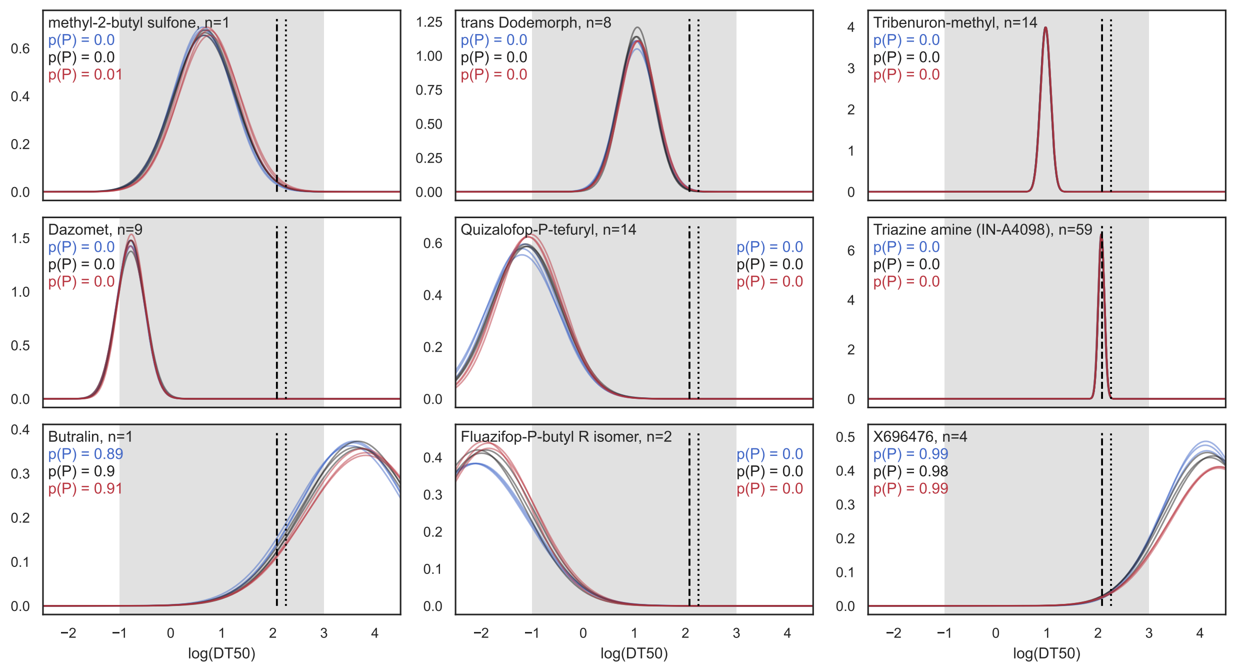

Figure S5: Varying prior parameter μ_{mean}. Each subplot shows the estimated half-life distribution (posterior mean μ_{mean} and its uncertainty μ_{std}) for a selected compound with n reported half-lives. Blue: μ_{mean}=0.5, black: μ_{mean}=1, red: μ_{mean}=1.5. The dashed and the dotted lines indicate the threshold values for P (120 days) and vP (180 d), respectively.

amine, fluazifop-P-buyl R isomer), the changes in the prior parameters did not affect p(P). For the remaining compounds, we observed a minimal effect of the prior parameters for those with few reported half-lives (<15). E.g., butralin has only one right-censored data point available, and its estimated probability of being persistent varies by maximum of 2 percent points when the parameters μ_{mean} or μ_{std} are in- or decreased by 0.5 log(days). Similarly, p(P) varies by a maximum of 1 percent points for the σ parameters when their value is modified by plus or minus 0.1. In another case, the persistence probability of trans dodemorph with 8 uncensored reported half-lives is modified by a maximum of 1 percent point when varying the σ parameters, but it is not affected by changes in the μ_{std} parameters. As a conclusion, the effect of varying prior parameters within reasonable ranges remains marginal and does not affect the overall assessment of a compound's persistence.

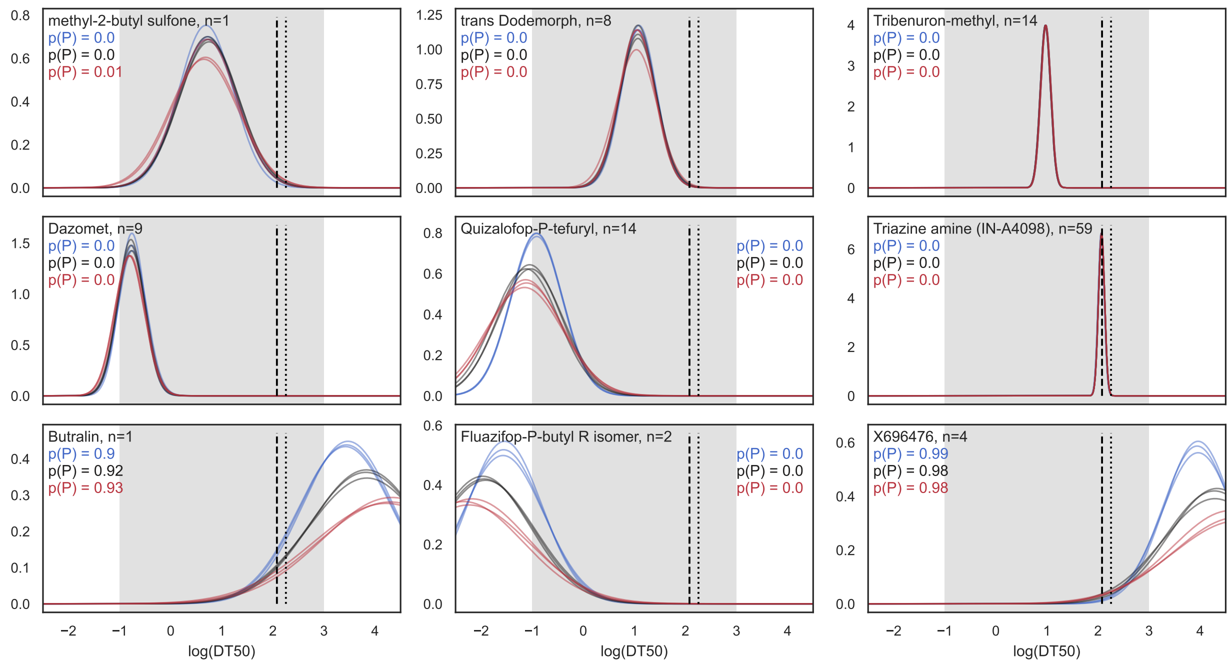

Figure S6: Varying prior parameter μ_{std}. Each subplot shows the estimated half-life distribution (posterior mean μ_{mean} and its uncertainty μ_{std}) for a selected compound with n reported half-lives. Blue: μ_{std}=1.5, black: μ_{std}=2, red: μ_{std}=2.5. The dashed and the dotted lines indicate the threshold values for P (120 days) and vP (180 d), respectively.

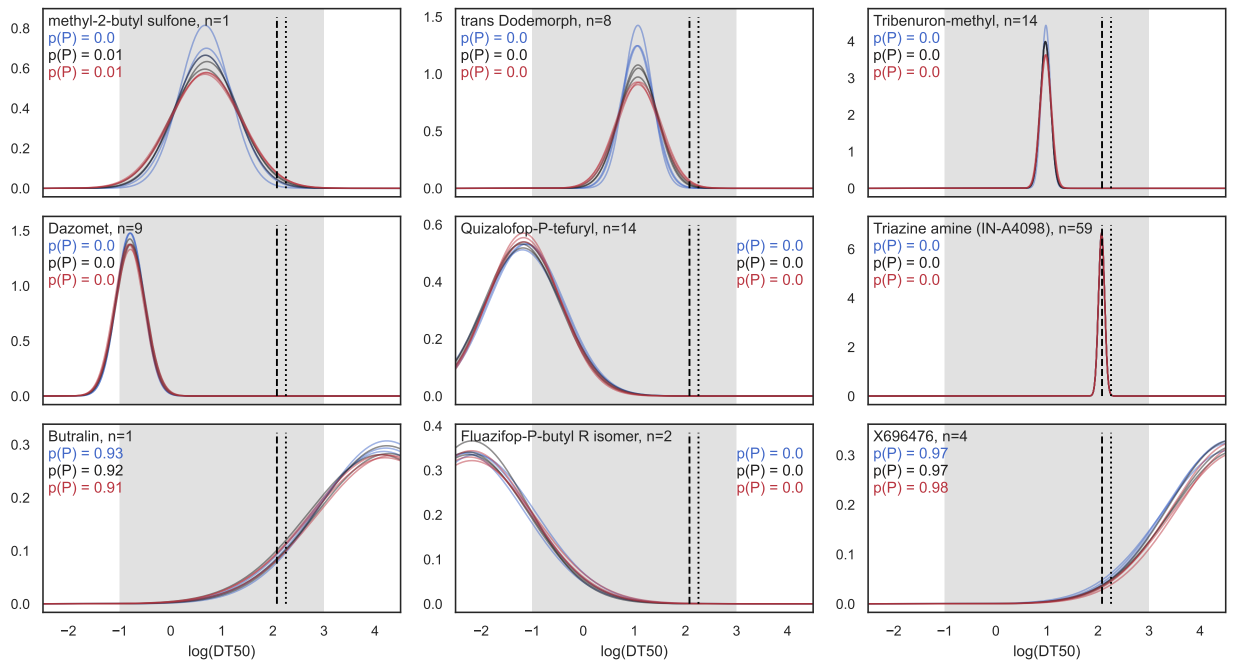

Figure S7: Varying prior parameter σ_{mean}. Each subplot shows the estimated half-life distribution (posterior mean μ_{mean} and its uncertainty μ_{std}) for a selected compound with n reported half-lives. Blue: σ_{mean}=0.3, black: σ_{mean}=0.4, red: σ_{mean}=0.5. The dashed and the dotted lines indicate the threshold values for P (120 days) and vP (180 d), respectively.

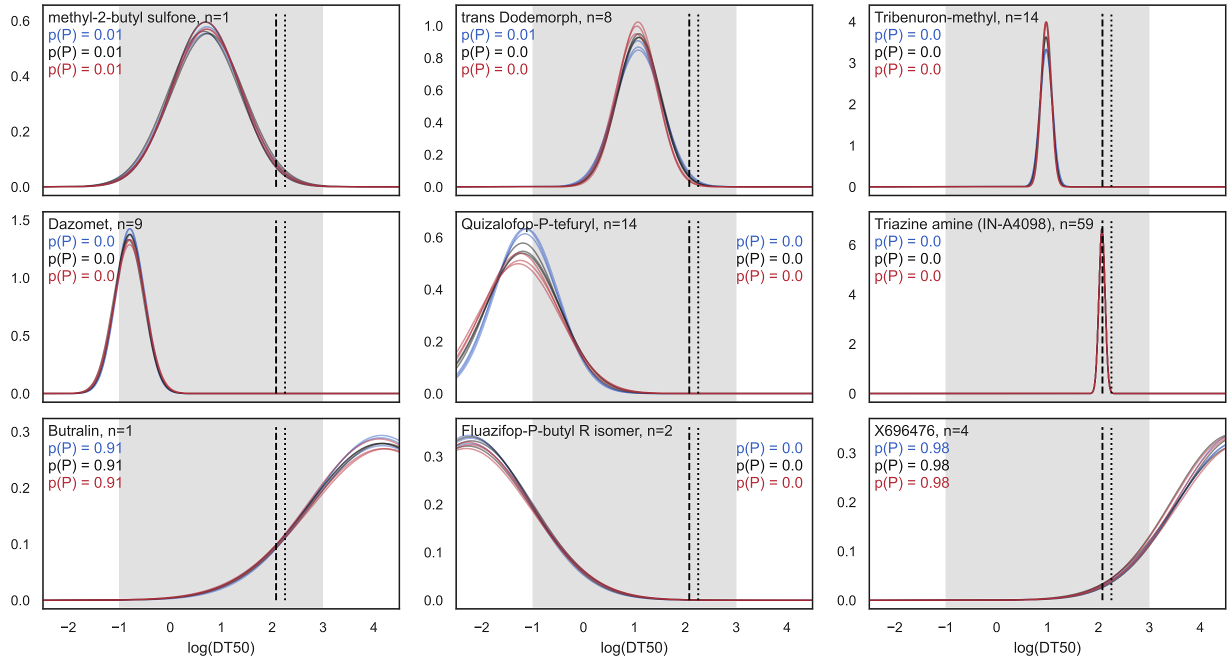

Figure S8: Varying prior parameter σ_{std}. Each subplot shows the estimated half-life distribution (posterior mean μ_{mean} and its uncertainty μ_{std}) for a selected compound with n reported half-lives. Blue: σ_{std}=0.3, black: σ_{std}=0.4, red: σ_{std}=0.5. The dashed and the dotted lines indicate the threshold values for P (120 days) and vP (180 d), respectively.

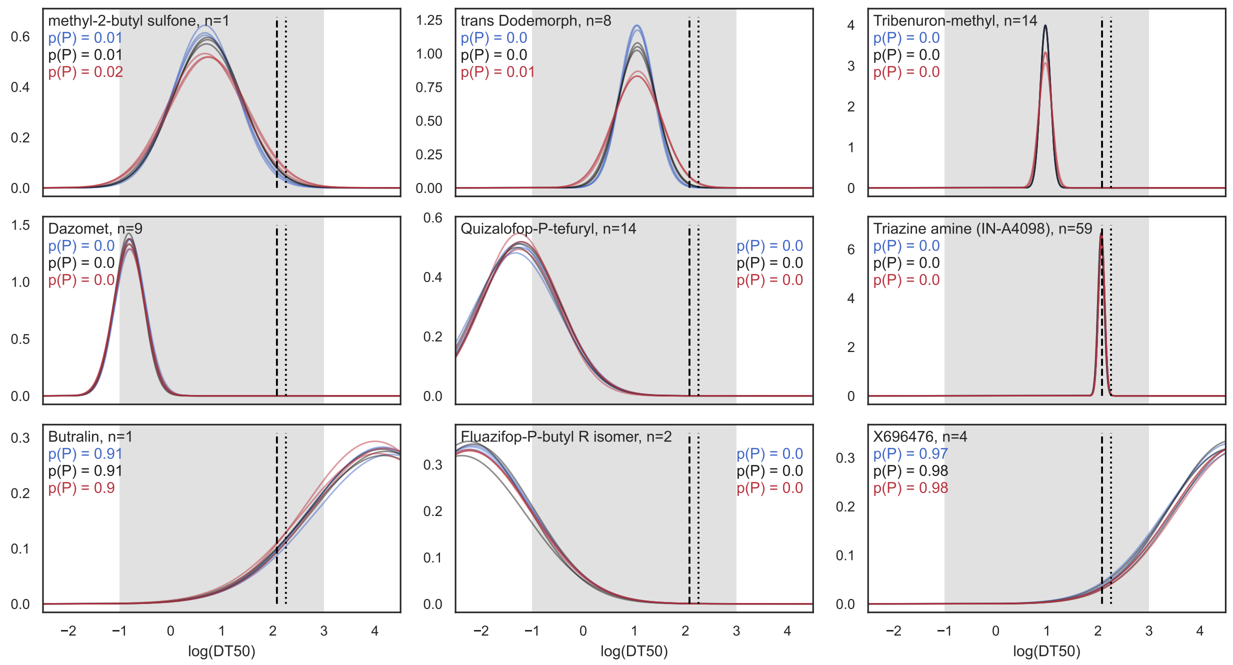

Figure S9: Varying prior parameter σ_{min}. Each subplot shows the estimated half-life distribution (posterior mean μ_{mean} and its uncertainty μ_{std}) for a selected compound with n reported half-lives. Blue: σ_{min}=0.1, black: σ_{min}=0.2, red: σ_{min}=0.3. The dashed and the dotted lines indicate the threshold values for P (120 days) and vP (180 d), respectively.

S2 Model Selection and Hyperparameter Optimization

Table S2: Evaluated combinations of model type, molecular feature representation, and dimensionality reduction strategy. All combinations were evaluated using nested cross-validation.

Model	Feature set	Dimensionality reduction
RF	PaDEL descriptors	None
RF	MACCS keys	None
RF	RDKit fingerprints	None
RF	RDKit descriptors	None
RF	Avalon fingerprints (avalonfps)	None
RF	enviPath-triggered rules (eptrig)	None
RF	Combined features	None
GPR	PaDEL descriptors	None
GPR	PaDEL descriptors	PCA
GPR	MACCS keys	None
GPR	MACCS keys	PCA
GPR	RDKit fingerprints	None
GPR	RDKit fingerprints	PCA
GPR	RDKit descriptors	None
GPR	RDKit descriptors	PCA
GPR	Avalon fingerprints (avalonfps)	None
GPR	Avalon fingerprints (avalonfps)	PCA
GPR	enviPath-triggered rules (eptrig)	None
GPR	enviPath-triggered rules (eptrig)	PCA
GPR	Combined features (all)	None
GPR	Combined features (all)	PCA

For the GPR models, we tested three commonly used kernels, namely Radial Basis Function (RBF), Matérn kernels with ν = 0.5, 1.5, 2.5, and the Rational Quadratic Kernel. Specific hyperparameters were optimized internally through the maximization of the marginal likelihood during training.[S3] Random Forest hyperparameters included the number of trees, maximum depth of each tree, the fraction of samples drawn from the dataset with replacement for each tree and the minimum number of samples required to split an internal node (Table S3).

Table S3: Hyperparameter configurations evaluated for Random Forest (RF) and Gaussian Process Regression (GPR) models. All kernels were multiplied with a constant kernel with bounds (1e-3, 1e3).

Model	Component	Hyperparameter	Values Tested
RF	Regressor	$n_{estimators}$	10, 50, 100
		max_depth	None, 5, 10, 20
		max_samples	0.5, 0.7, 0.9, None
		min_samples_split	2, 5, 10
GPR	Regressor	normalize_y	True
		$n_{restarts\_optimizer}$	0, 1, 2
	Kernel	Radial Basis Function	lengthscale bounds = (1e-3, 1e3)
		Matérn ν	0.5,1.5,2.5
			lengthscale bounds = (1e-3, 1e3)
		Rational Quadratic	$\alpha = 1.0$
			lengthscale bounds = (1e-3, 1e3)
	Feature reduction	PCA components	33, 34, 35, 36, 37, 38, 39, 40

Nested cross-validation was used to obtain an unbiased estimate of model performance, and to tune hyperparameters for each combination of model type, molecular feature representation, and dimensionality reduction listed in Table S2. First, the data set was split into five outer folds. In each outer iteration, one fold was held out as an outer test set, while the remaining four folds were used as the outer training set. Within each outer training set, a five-fold inner cross-validation was performed for model selection. A grid search over all hyperparameter combinations (Table S3) was conducted. Each candidate configuration was then evaluated over the inner training data and performance was tracked using R^2. For each outer iteration, the configuration achieving the highest mean inner cross validation R^2 was selected. This configuration was then used to fit the outer training set and was evaluated on the corresponding outer test fold. This process was repeated for all five outer folds, producing five independent outer test R^2 scores. In a majority vote fashion, the final hyperparameters for the final model were chosen as those most frequently selected across the five outer folds (Table S4). The GPR and RF were implemented using scikit learn.[S4]

Table S4: Best-performing hyperparameter configurations for Gaussian Process Regression (GPR) and Random Forest (RF) models. GPR kernel is multiplied by ConstantKernel with bounds (1e-3, 1e3).

**Model**	**Component**	**Hyperparameter**	**Best Value**
RF	Regressor	$n_{estimators}$	100
		max_depth	None
		max_samples	None
		min_samples_split	5
GPR	Regressor	normalize_y	True
		$n_{restarts\_optimizer}$	0
	Kernel	Matérn	$\nu = 0.5$
			lengthscale bounds = (1e-3, 1e3)
	Feature reduction	PCA components	no feature reduction

S3 Model evaluation

S3.1 Expected Calibration Error (ECE)

The ECE summarizes the mean deviation between nominal confidence levels and the empirical fractions of observed values falling within the corresponding confidence intervals. In a perfectly calibrated model, at a 95 % confidence level, it is expected that the confidence intervals, defined by the prediction uncertainty, contain the true observed values for 95% of the predictions. The ECE is the averaged deviation of the expected vs empirical fractions over all defined confidence levels. A Low ECE indicates good uncertainty calibration.

We have N predictions with predicted values $\hat{y}_i$ ($\log \mathrm{DT}_{50,\mathrm{pred}}$), prediction standard deviations $\hat{\sigma}_i$ ($\log \mathrm{DT}_{50,\mathrm{std}}$), and bayesian inferred mean estiamtes y_i. The symmetric Gaussian confidence intervals at nominal confidence level $\alpha \in \mathcal{A}$ are defined as:

$$\hat{y}_i \pm z_\alpha \hat{\sigma}_i, \qquad z_\alpha = \Phi^{-1}\left(\tfrac{1+\alpha}{2}\right),$$

where Φ^{-1} is the inverse cumulative distribution function of the standard normal distribution.

The empirical coverage corresponding to α is

$$\hat{C}(\alpha) = \frac{1}{N} \sum_{i=1}^{N} \mathbf{1}(|y_i - \hat{y}_i| \leq z_\alpha \hat{\sigma}_i)\,,$$

$\mathbf{1}(\cdot)$ denotes the indicator function. The Expected Calibration Error (ECE) is defined then defined as[S5,S6]

$$\text{ECE} = \frac{1}{|\mathcal{A}|} \sum_{\alpha \in \mathcal{A}} \left| \alpha - \hat{C}(\alpha) \right|.$$

Perfect calibration corresponds to $\hat{C}(\alpha) = \alpha$ for all α, yielding ECE $= 0$.

S3.2 Expected Normalized Calibration Error (ENCE)

The ENCE evaluates whether residuals scale appropriately with the predicted uncertainties. It describes the mean absolute deviation between observed error and prediction uncertainty across batches. It relies on standardized errors, meaning residuals divided by their predicted standard deviation. To compute it, predictions are ordered by increasing uncertainty and binned into $\sqrt{N}$ batches.[S7] Analogous to ECE, a lower ENCE means better uncertainty calibration. To evaluate whether residuals scale with prediction uncertainties, predictions are sorted by increasing $\hat{\sigma}_i$ and binned into $B = \sqrt{N}$[S7]batches $\{\mathcal{D}_b\}_{b=1}^{B}$. For each batch, the root-mean-square error (RMSE) and root-mean uncertainty (RMU) are calculated as:

$$\text{RMSE}_b = \sqrt{\frac{1}{|\mathcal{D}_b|} \sum_{i \in \mathcal{D}_b} (y_i - \hat{y}_i)^2}$$

$$\text{RMU}_b = \sqrt{\frac{1}{|\mathcal{D}_b|} \sum_{i \in \mathcal{D}_b} \hat{\sigma}_i^2}$$

The expected Normalized Calibration Error (ENCE) is then defined as[S5]

$$\text{ENCE} = \frac{1}{B} \sum_{b=1}^{B} \frac{|\text{RMSE}_b - \text{RMU}_b|}{\text{RMU}_b}$$

ENCE = 0 corresponds to perfect calibration.

S3.3 Distance-Uncertainty Relationship

We analyzed the relationship between prediction uncertainty and structural dissimilarity to the training data. The analysis follows the distance-based uncertainty framework prposed by Yin *et al.* (2023)[S6] and as applied by von Borries *et al.* (2025).[?] The structural dissimilarity was quantified as the average Tanimoto distance between each test compound and its five nearest neighbors in the training set based on Morgan fingerprints. Analougously to ENCE, predictions were sorted by increasing prediction uncertainty and grouped into batches. For each batch, the RMU and the mean Tanimoto distance were compouted. The relationship was then assessed using Pearson's r and Spearmans ρ.

S4 Model Performance

S4.1 Model cross-validation

Table S5: *Model Results.* Predictive performance, uncertainty calibration, and distance–uncertainty correlations for all evaluated model–feature combinations. Results are reported for Random Forest (RF) and Gaussian Process Regression (GPR) models trained on different molecular descriptor sets, with and without principal component analysis (PCA) for dimensionality reduction. Model performance is quantified using the coefficient of determination (R^2) and the root-mean-square error (RMSE). Uncertainty calibration is evaluated using the expected calibration error (ECE) and the expected normalized calibration error (ENCE). The relationship between predicted uncertainty and distance from the training domain is assessed using Spearman's and Pearson's r correlation coefficients between predicted uncertainty and the average Tanimoto distance to the five nearest training compounds. All metrics are averaged across 5-fold nested cross-validation.

R2	RMSE	ECE	ENCE	Spearman's r	Pearson's r	model	features	reduction
0.28	0.81	5.00	15.80	0.92	0.90	RF	all	None
0.26	0.83	4.60	14.80	0.91	0.89	RF	padel	None
0.23	0.84	3.90	21.50	0.81	0.80	RF	rdkitfps	None
0.23	0.84	4.80	20.00	0.90	0.93	RF	rdkitdesc	None
0.26	0.83	1.30	23.80	0.92	0.87	RF	avalonfps	None
0.23	0.85	3.10	20.50	0.94	0.93	RF	maccs	None
0.17	0.88	15.60	124.00	-0.36	-0.33	RF	eptrig	None
0.26	0.83	1.60	22.30	0.92	0.91	GPR	all	None
0.24	0.84	1.40	23.60	0.85	0.86	GPR	all	pca
0.32	0.79	1.20	20.50	0.98	0.92	GPR	padel	None
0.30	0.80	1.40	22.40	0.94	0.89	GPR	padel	pca
0.23	0.84	1.50	26.20	0.87	0.72	GPR	rdkitfps	None
0.21	0.86	1.30	24.30	0.70	0.58	GPR	rdkitfps	pca
0.28	0.82	1.20	22.40	0.58	0.69	GPR	rdkitdesc	None
0.27	0.82	1.00	23.20	0.40	0.56	GPR	rdkitdesc	pca
0.26	0.83	0.80	20.90	0.87	0.85	GPR	avalonfps	None
0.24	0.84	1.10	21.00	0.84	0.77	GPR	avalonfps	pca
0.26	0.83	1.50	25.30	0.93	0.75	GPR	maccs	None
0.25	0.83	1.80	26.10	0.94	0.77	GPR	maccs	pca
0.12	0.90	19.00	422.00	0.38	0.38	GPR	eptrig	None
0.11	0.90	19.10	422.40	0.35	0.37	GPR	eptrig	pca

Inspection of the parity plot (Figure S10, upper left) shows that the prediction for the bulk of compounds with μ_{mean} between 0 and 2.5 log(days) lie reasonably close to the x=y line,

whereas those at the extremes (low and high persistence) exhibit the largest errors. The low-end tail is over-predicted and the high-end tail under-predicted. This pattern is mirrored in the RF results (Figure S11, upper left). The models are most likely biased towards the mean of the target value distribution due to the target value distribution being roughly normally distributed with a mean around 1.2 log(days) (Figure S2). Taken together, while the models can capture a very broad trend in soil μ_{mean}, their quantitative accuracy remains limited. Including uncertainty awareness should account for these residuals.

In addition to the predicted mean half-life $DT_{50,\mathrm{pred}}$, the model uncertainty estimate $DT_{50,\mathrm{std}}$ is captured by the GPR around each $DT_{50,\mathrm{pred}}$. The uncertainty estimates for the best GPR model range from 0.21 to 0.91 log(days) (Figure S10, upper right). RF, in contrast, produces uncertainty estimates from the tree ensemble variance that span a broader range from 0.26 to 1.58 log(days) (Figure S11, upper right). For both models, the $DT_{50,\mathrm{std}}$ displays a clear dependence on the proximity of test compounds to the training set (Figures S10 and S11, lower left). This is especially pronounced for GPR (Spearman's $r= 0.98$; Pearson's $r = 0.92$) and also clearly visible for RF (Spearman's $r= 0.92$; Pearson's $r = 0.90$). In other words, uncertainty tends to be smallest for compounds that are within well represented regions of the training set's chemical space. As distance increases, so does $DT_{50,\mathrm{std}}$, indicating that remoteness from known chemical space is a primary driver for model uncertainty. However, in the case of GPR, high predictive uncertainty can arise from an additional effect beyond distance alone. If a test compound lies near a region where the training compounds themselves have high $DT_{50,\mathrm{std}}$, the model will propagate that uncertainty into its predictions. Visually, this can be confirmed looking at the fitted training uncertainties (Figure S12). Clearly, the fitted uncertainty reflects the estimated mean uncertainty μ_{std} in the GPR model. For RF, the $DT_{50,\mathrm{std}}$ are largely independent of μ_{std}.

The confidence calibration reveals that the GPR model (ECE = 1.1%, Figure S10, middle left) and the RF model (ECE = 4.8%, Figure S11, middle left) are well calibrated. There is a very slight under-confidence in the case of the RF model, which we deem to be negligible.

Good confidence calibration means that credible intervals can be used reliably in downstream decision-making. When analyzing the error-calibration plot for GPR (Figure S10, middle right), we see a trend between RMSE and model uncertainty for GPR (ENCE = 20.5%, Spearman's r= 0.42; Pearson's r = 0.59) and similar for the RF (ENCE = 14.8%, Spearman's r = 0.54; Pearson's r = 0.64, Figure S11, middle right)). This highlights that both models reflect their predictive performance by means of the model uncertainty. Interestingly, RF shows a stronger correlation between uncertainty and prediction error than GPR. Yet this does not mean it provides a more accurate estimation of uncertainty. In contrast to RF, the GPR uncertainty range is more compact and partly constrained by the kernel and hyperparameters, which bound the uncertainties. This narrower spread reduces the strength of correlation compared to RF.

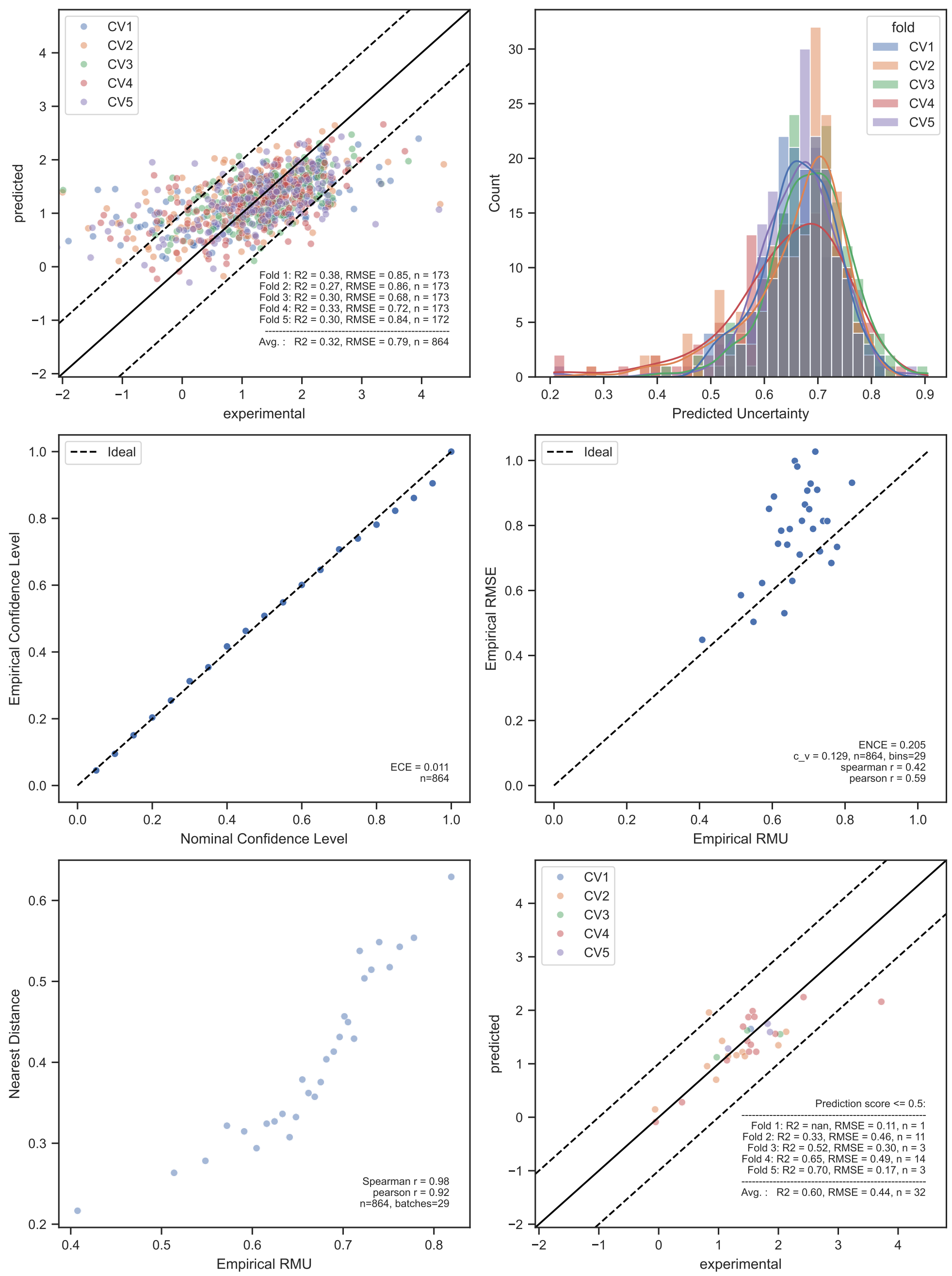

Figure S10: **Performance and uncertainty analysis of the Gaussian Process Regression (GPR) model trained on PaDEL descriptors using five-fold cross-validation.** Top left: parity plot of predicted versus experimental soil half-lives (log DT_{50}). Top right: distribution of predictive uncertainties ($DT_{50,std}$) across folds. Middle left: confidence calibration plot comparing nominal and empirical coverage. Middle right: relationship between prediction error and predicted uncertainty. Bottom left: dependence of predictive uncertainty on chemical distance to the training set. Bottom right: parity plot of predictions with a prediction score below 0.5. Results are aggregated over all outer cross-validation folds.

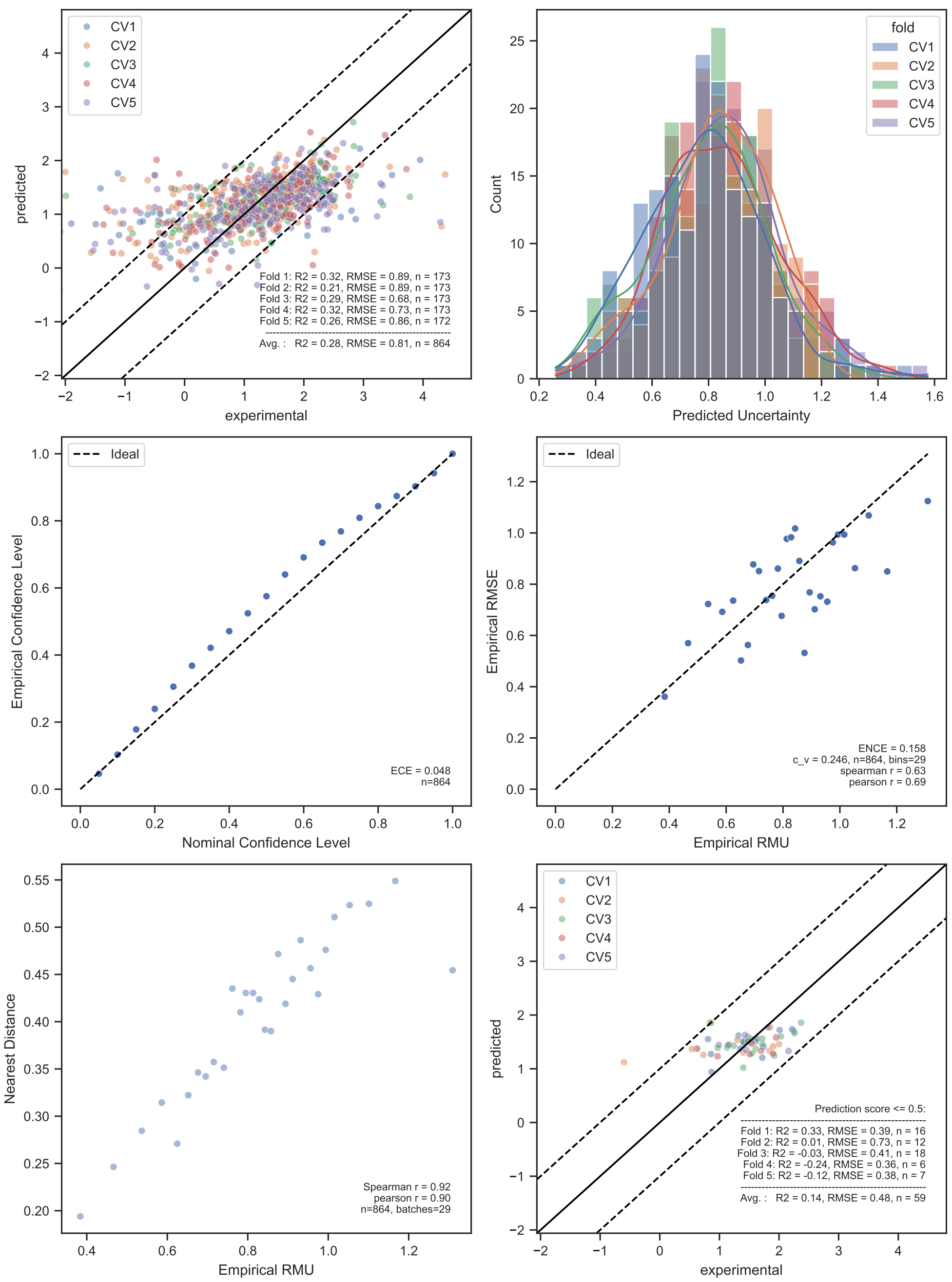

Figure S11: **Performance and uncertainty analysis of the Random Forest (RF) model trained on PaDEL descriptors using five-fold cross-validation.** Top left: parity plot of predicted versus experimental soil half-lives (log DT_{50}). Top right: distribution of predictive uncertainties derived from ensemble variance across folds. Middle left: confidence calibration plot comparing nominal and empirical coverage. Middle right: relationship between prediction error and predicted uncertainty. Bottom left: dependence of predictive uncertainty on chemical distance to the training set. Bottom right: parity plot of predictions with a prediction score below 0.5. Results are aggregated over all outer cross-validation folds.

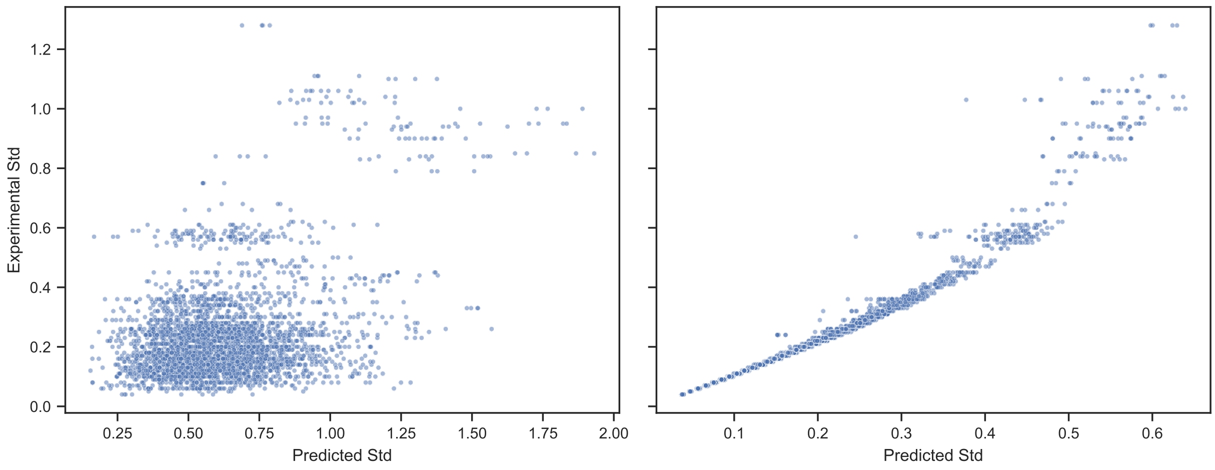

Figure S12: **Training set fitted uncertainty (predicted) vs μ_{std} (experimental std)** for RF (left) and GPR (right).

S4.2 Validation on external dataset

We collected a 99 soil half-lives for 25 substances from the PREMIER DAS[1], the ECHA CHEM portal[2], and from EFSA[3]. We used Bayesian inference with the same prior assumptions as for the training dataset to calculate representative half-life distributions for each substance. We then applied our model (PEPPER), the VEGA primary biotransformation half-life prediction module,[S8] and EPISuite^{TM} BIOWIN4 (primary biotransformation)[S9] to the 25 compounds and compared the predicted half-lives to the Bayesian-inferred mean logDT50.

PEPPER outperforms VEGA and BIOWIN4 in terms of R^2 and RMSE. VEGA classifies all its predictions as "LOW reliability". Moreover, the 95% confidence intervals of our model predictions always overlap with the range of +/- 1 log(days) surrounding the true mean logDT50, with the exception of 8-methyldecan-2-yl propanoate. This very fast-degrading compounds was tested under different test conditions (shorter experiments with narrower sampling times) than the compounds in the training data, and its reported half-lives lie

[1]https://premier.marionegri.it/
[2]https://chem.echa.europa.eu/
[3]https://www.efsa.europa.eu/en/calls/consultations

ID	Compound name	source	logDT50 mean	logDT50 std	PEPPER	BIOWIN4	VEGA	PEPPER std
1	Regorafenib	PREMIER DAS	2.24	0.59	1.45	2.15	2.07	0.74
2	Atovaquone	PREMIER DAS	-0.88	1.12	1.20	1.82	3.36	0.70
3	Dolutegravir sodium	PREMIER DAS	4.32	0.93	1.85	1.41	1.53	0.78
4	Mirtazapine	PREMIER DAS	2.24	0.19	2.14	2.42	2.00	0.71
5	Taranabant	PREMIER DAS	3.78	1.10	1.68	2.76	3.36	0.74
6	Vorapaxar sulphate	PREMIER DAS	2.70	0.20	1.21	1.32	1.35	0.74
7	Ceritinib	PREMIER DAS	2.08	0.35	1.51	2.65	2.07	0.76
8	Nilotinib	PREMIER DAS	1.59	0.36	1.90	2.49	1.36	0.82
9	Panobinostat lactate	PREMIER DAS	-0.02	0.28	1.30	1.77	0.89	0.75
10	Orlistat	PREMIER DAS	0.73	0.23	0.60	0.18	1.36	0.83
11	Vemurafenib	PREMIER DAS	4.33	0.98	1.94	2.16	2.07	0.76
12	Elbasvir	PREMIER DAS	4.22	0.95	1.50	1.47	1.36	0.97
13	Decamethyltetrasiloxane	ECHA	0.97	0.28	1.49	1.55	1.37	0.92
14	EC 10081-67-1	ECHA	1.96	0.22	1.73	2.34	1.83	0.77
15	EC 214-946-9	ECHA	2.13	0.26	1.44	2.00	1.36	0.74
16	Octamethyltrisiloxane	ECHA	0.68	0.28	1.43	1.39	1.37	0.89
17	EC 221-374-3	ECHA	-0.12	0.39	1.27	1.80	0.86	0.68
18	EC 225-625-8	ECHA	2.91	0.27	1.37	1.62	1.85	0.83
19	Tert-butyl methyl ether	ECHA	1.95	0.38	1.01	1.32	1.03	0.75
20	Inpyrfluxam	EFSA	2.02	0.22	2.39	1.62	1.36	0.61
21	Fluoxapiprolin	EFSA	1.42	0.16	1.64	2.30	2.34	0.78
22	Cinnamaldehyde	EFSA	-2.08	0.87	0.26	0.88	1.10	0.75
23	Cinnamic acid	EFSA	0.24	0.24	0.23	0.63	1.10	0.66
24	Beflubutamid	EFSA	1.48	0.43	0.83	1.60	0.89	0.66
25	8-methyldecan-2-yl propanoate	EFSA	-2.10	0.87	0.66	1.04	1.41	0.67

Table S6: **Comparison of PEPPER, BIOWIN4, and VEGA predictions.** logDT50 mean: logDT50 [log days] as estimated by Bayesian inference from experimental data points, logDT0 std: Uncertainty of the estimated mean as a standard deviation, PEPPER: logDT50 predicted by PEPPER, BIOWIN4: logDT50 obtained from transforming the BIOWIN4 output for primary biotransformation using the equation postulated by Arnot *et al.* (2005),[S1] VEGA: logDT0 predicted by VEGA version 1.2.6, PEPPER std: uncertainty of the logDT50 predicted by PEPPER as a standard deviation.

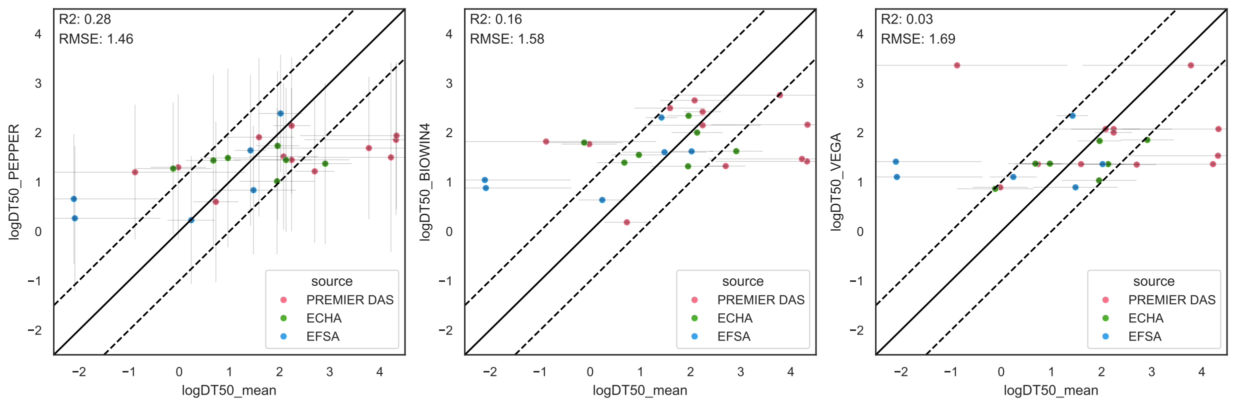

Figure S13: Comparison of PEPPER, BIOWIN4, and VEGA prediction performance on the external data set. Error bars represent the 95% confidence intervals for the logDT50 uncertainty of the mean on the y-axis, and the prediction uncertainty for PEPPER predictions on the x-axis.

beyond the dynamic range of the training data, ranging from -1 to 3 log(days). Cinnamaldehyde and cinnamatic acid were tested under similar conditions, hence we would expect our model to assign high uncertainties to the predicted half-lives of these compounds, especially if their reported half-lives lie beyond the dynamic range. It is important to note that among the tested models, our model still makes the most accurate prediction for the two compounds with the shortest reported half-lives (8-methyldecan-2-yl propanoate and cinnamaldehyde). Furthermore, it is important to note that our model performs very similarly on the external validation data set ($R^2 = 0.28$) as compared to the internal cross-validation folds ($R^2 = 0.32$), indicating a robust model performance on unseen data.

S5 Application 1: Predicting half-lives for pesticide TPs without kinetic data

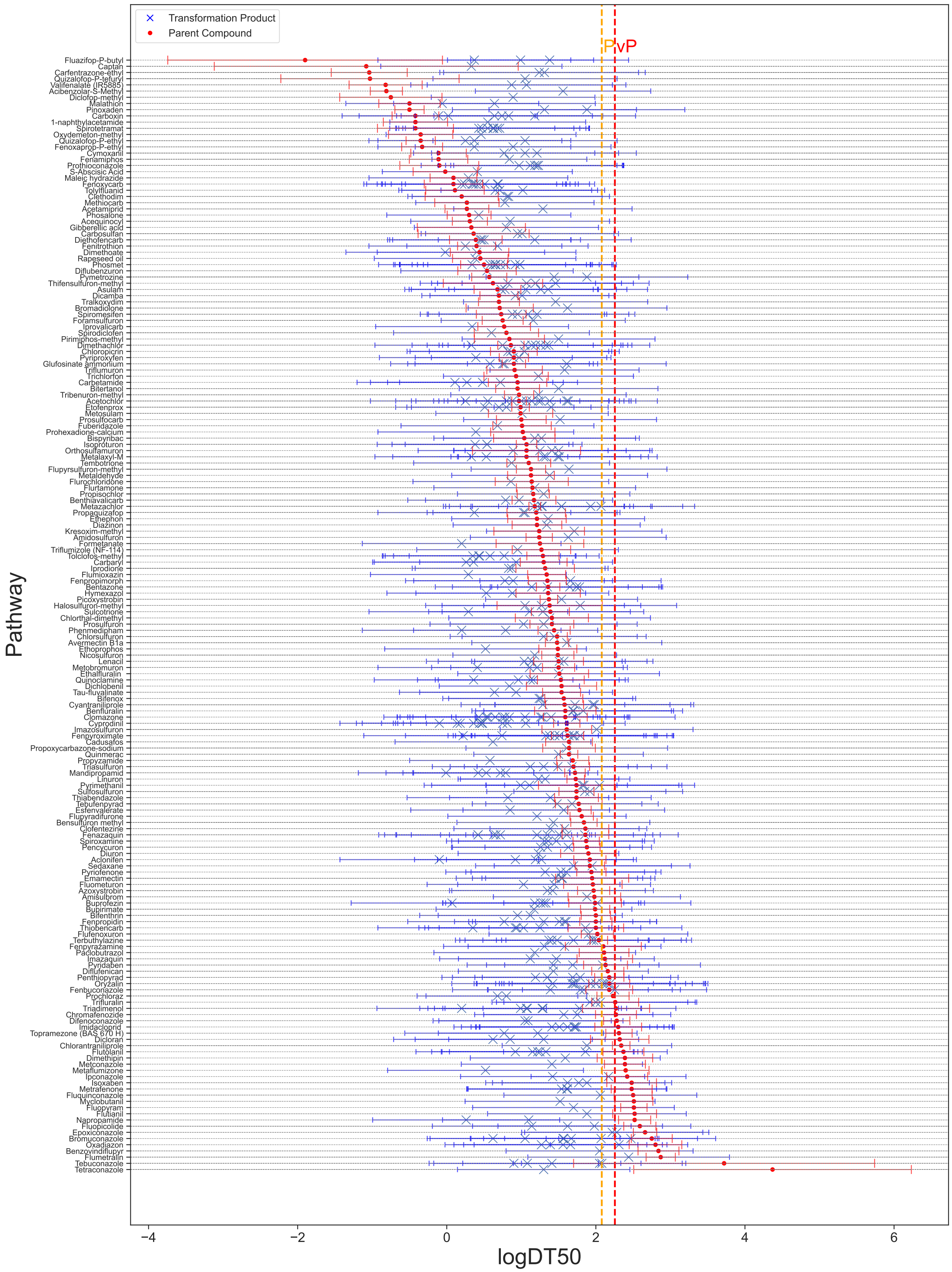

Figure S14: The Bayesian estimated μ_{mean} values for parent compounds (red points) and their associated transformation products with predicted values $DT_{50,\text{pred}}$ (blue cross) filtered to $DT_{50,\text{std}} \leq 0.7$ log days. The horizontal bars are the 95 % intervals of the parent compounds (μ_{std}) and TPs ($DT_{50,\text{std}}$). Vertical dashed lines indicate the REACH persistence thresholds for persistent (P) and very persistent (vP) classification.

Predicted mean $\log \mathrm{DT}_{50,\mathrm{pred}}$, associated uncertainty $\log \mathrm{DT}_{50,\mathrm{std}}$, and persistence class probabilities for all transformation products can be found in the external file Predictions_pesticide_TPs.csv.

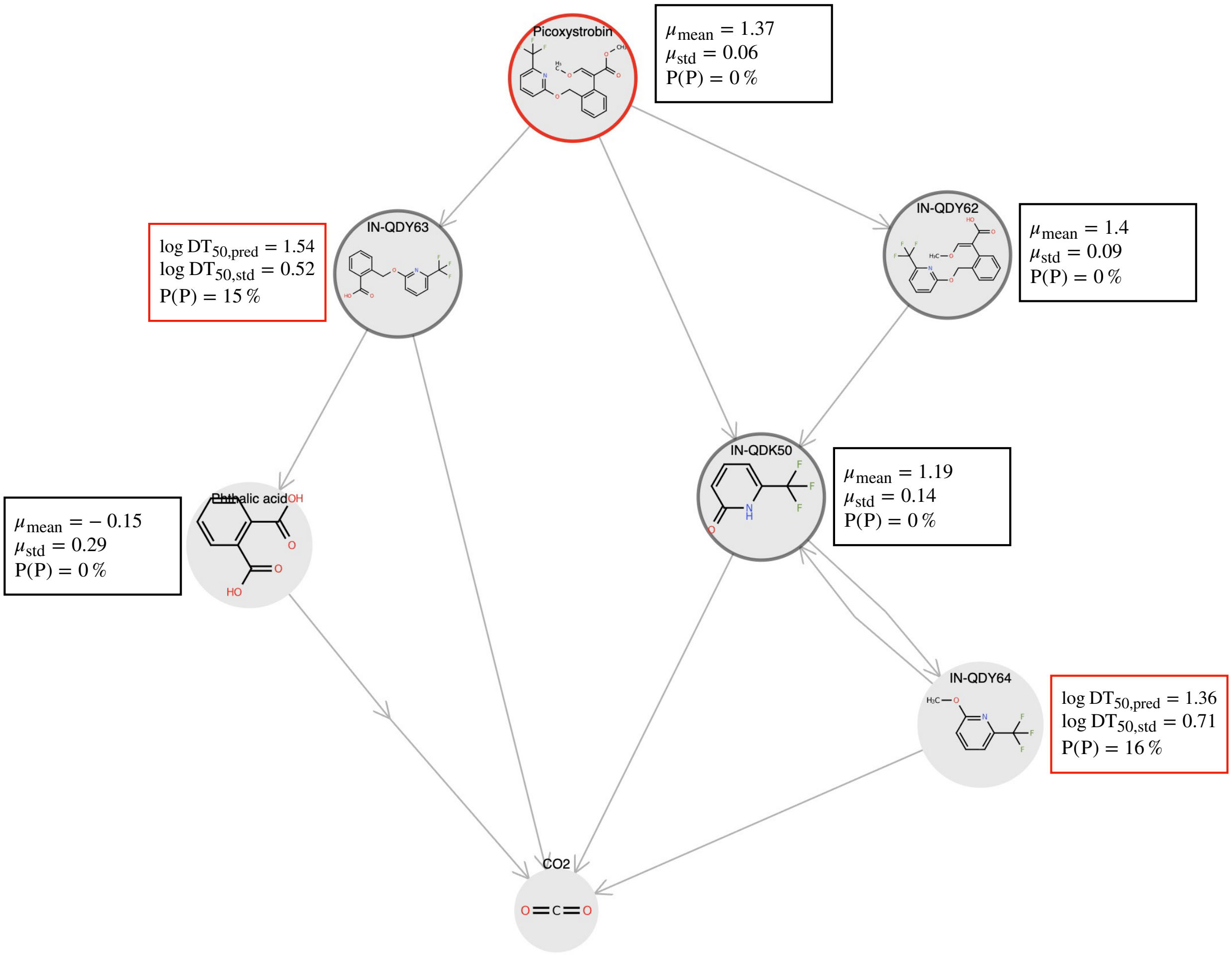

Figure S15: Picoxystrobin pathway in enviPath with reported (black box) and predicted (red boxes) half-lives, prediction uncertainties ($\log \mathrm{DT}_{50,\mathrm{std}}$), and persistence probabilities (p(P)). The red circle indicates the parent compound. $\log \mathrm{DT}_{50,\mathrm{pred}}$: predicted log half-life; $\log \mathrm{DT}_{50,\mathrm{std}}$: prediction log half-life uncertainty; μ_{mean}: Bayesian inferred mean log half-life; μ_{mean}: Bayesian inferred mean log half-life uncertainty

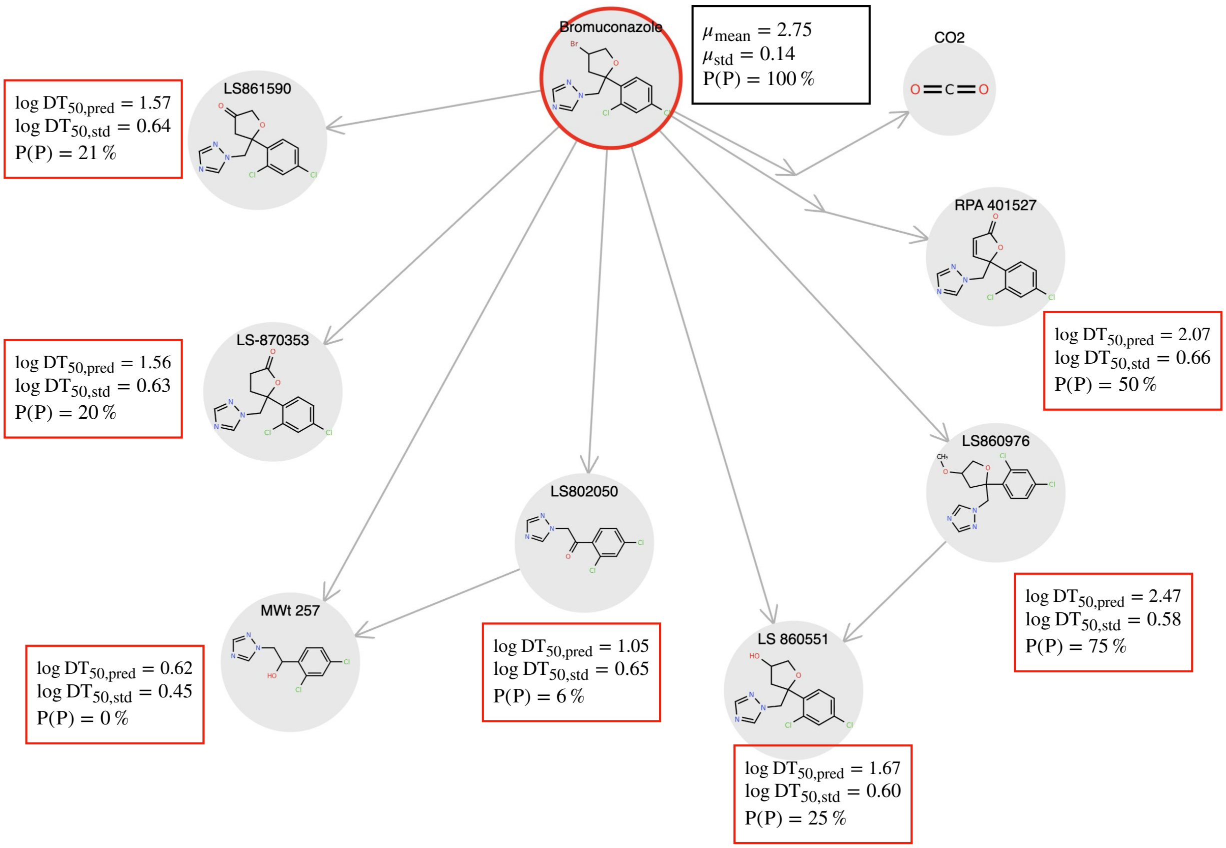

Figure S16: Bromuconazole pathway in enviPath with reported (black box) and predicted (red boxes) half-lives, prediction uncertainties ($\log DT_{50,std}$), and persistence probabilities (p(P)). The red circle indicates the parent compound. $\log DT_{50,pred}$: predicted log half-life; $\log DT_{50,std}$: prediction log half-life uncertainty; μ_{mean}: Bayesian inferred mean log half-life; μ_{mean}: Bayesian inferred mean log half-life uncertainty

Table S7: Overview on transformation products in EAWAG-SOIL with and without reported half-lives and organized by minor/major classification.

	minor	major	mixed	no information	total
total TPs	94	61	5	1220	1380
no reported DT_{50}	62	1	5	751	819
reported DT_{50}	32	60	0	469	561

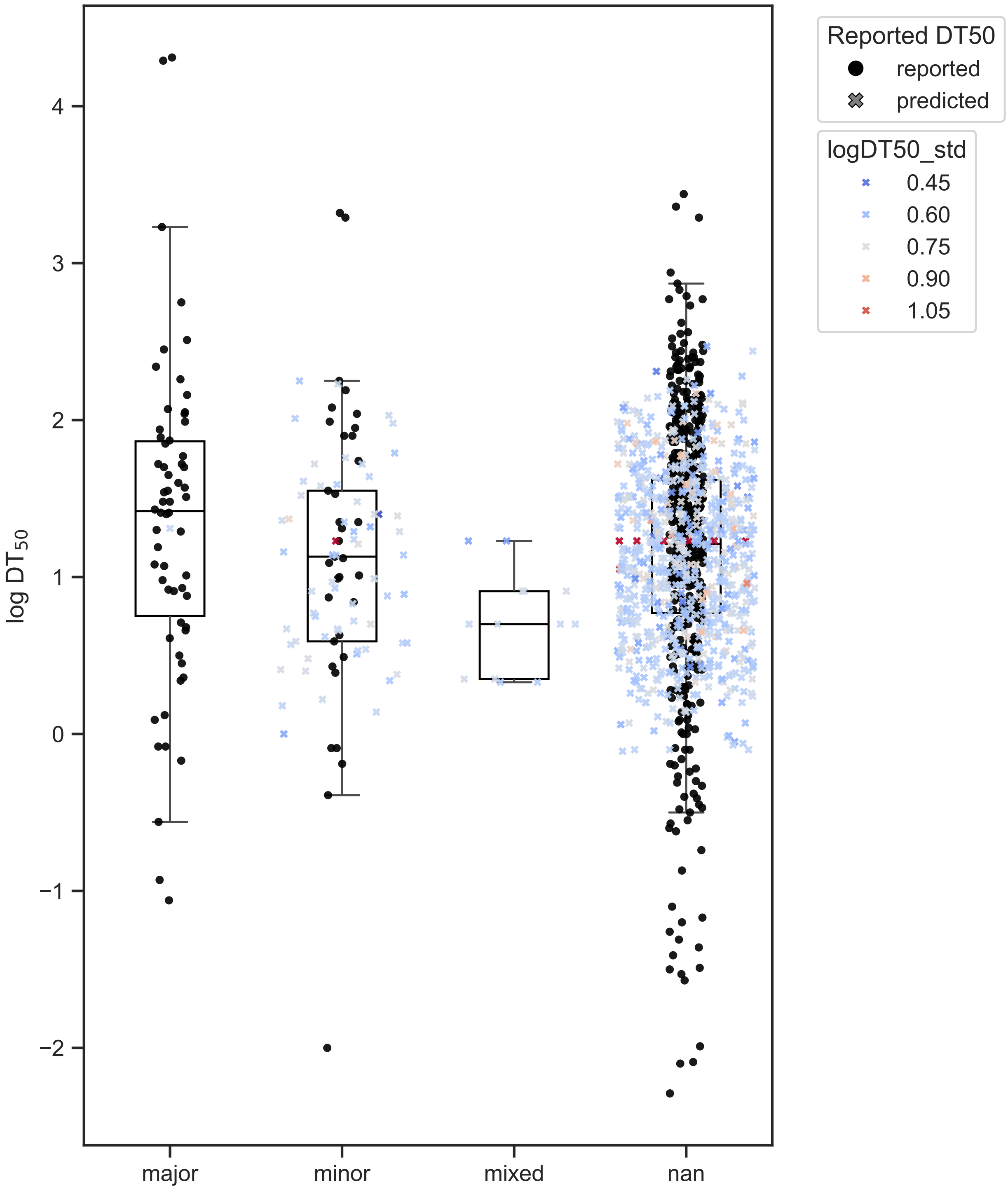

Figure S17: **Distribution of reported (dots) and predicted (crosses) biotransformation half-lives for different TP importance classifications.** The prediction confidence is indicated as $\log DT_{50,std}$ on a blue-red color scale. TPs classified as "mixed" are assigned "minor" and "major" in different experiments or pathways. TPs classified as "nan" have no TP importance assigned. The boxplots are derived from the sum of reported and predicted $\log DT_{50}$.

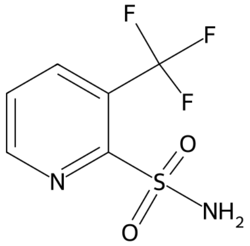

TP: TPSA (SSRE-001)
Pathway: Flazasulfuron
$\mu_{\text{mean}} = 3.32$
$\mu_{\text{std}} = 0.50$
P(P) = 100 %

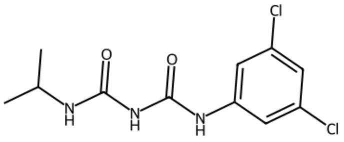

RP 36221
Pathway: Iprodione
$\mu_{\text{mean}} = 3.29$
$\mu_{\text{std}} = 0.39$
P(P) = 100 %

Figure S18: **Examples of minor TPs with high reported half-lives.** (top) TPSA (SSRE-001), a minor TP of the flazasulfuron pathway. (bottom) RP 36221, a minor TP of the iprodione pathway. The Bayesian inferred mean estimates (μ_{mean}), associated uncertainty (μ_{std}) and probability of persistence (p(P)) are reported, illustrating that a TP classified as minor does not necessarily imply fast degradation.

S6 Application 2: Predicting half-lives for marketed chemicals

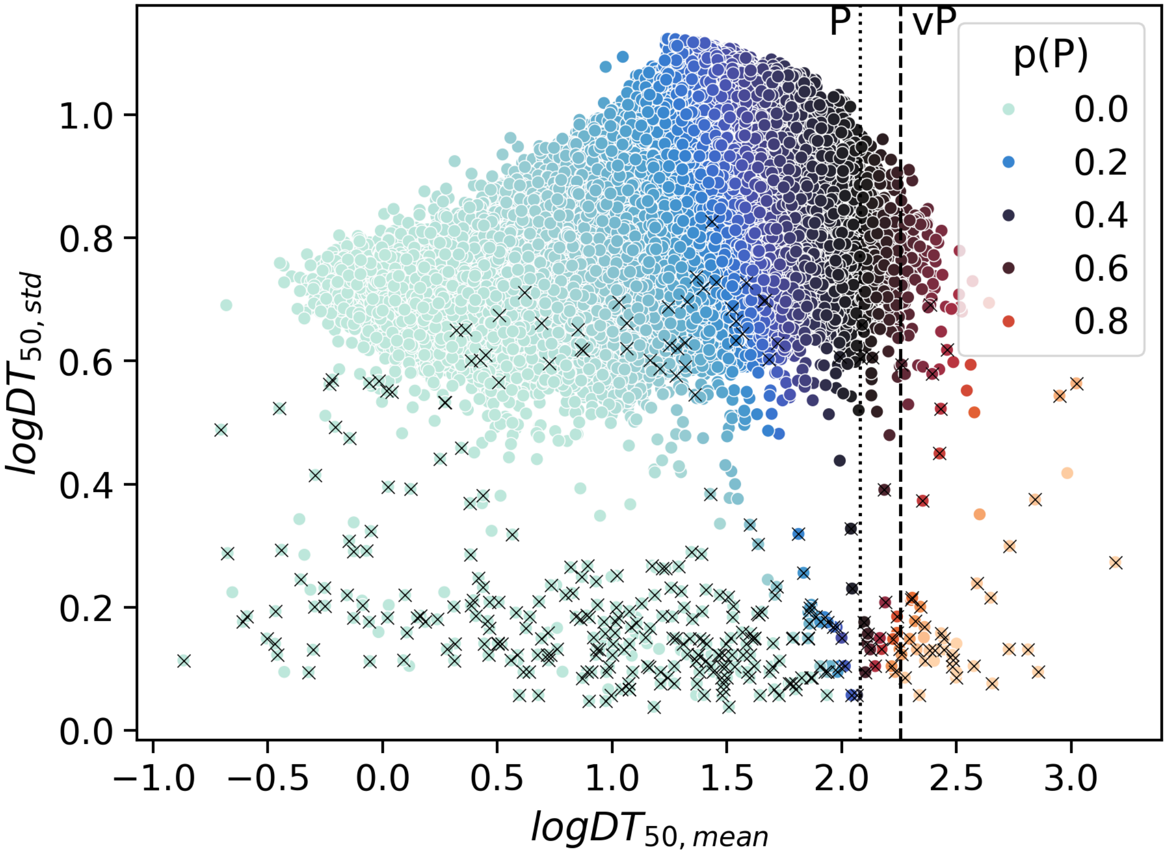

Figure S19: **Predicted mean** $\log \mathrm{DT}_{50,\mathrm{pred}}$ **and associated uncertainty** $\log \mathrm{DT}_{50,\mathrm{std}}$ **for 95,013 marketed chemicals in the ZeroPM database.** The colors indicate the predicted probability of each substance to be persistent (P), and crosses mark chemicals with experimental half-lives in the training data. The vertical dotted and dashed lines indicate the P and vP thresholds at 120 and 180 days, respectively.

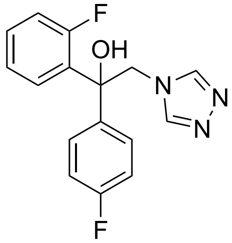

Flutriafol Impurity A
$\log DT_{50,pred} = 2.98$
$\log DT_{50,std} = 0.42$
p(P) = 0.98

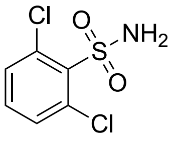

2,6-Dichlorobenzene sulfonamide
$\log DT_{50,pred} = 2.58$
$\log DT_{50,std} = 0.52$
p(P) = 0.83

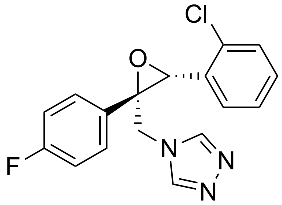

4-[[(2S,3R)-3-(2-chlorophenyl)-2-(4-fluorophenyl)oxiran-2-yl]methyl]-1,2,4-triazole
$\log DT_{50,pred} = 2.60$
$\log DT_{50,std} = 0.35$
p(P) = 0.93

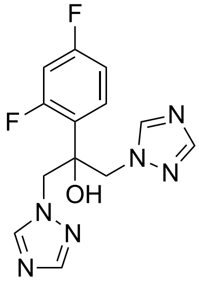

Fluconazole
$\log DT_{50,pred} = 2.52$
$\log DT_{50,std} = 0.68$
p(P) = 0.74

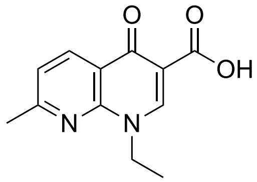

Nalidixic Acid
$\log DT_{50,pred} = 2.48$
$\log DT_{50,std} = 0.68$
p(P) = 0.72

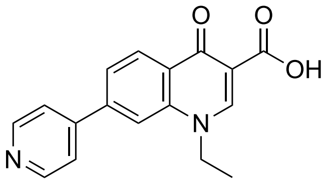

Rosoxacin
$\log DT_{50,pred} = 2.50$
$\log DT_{50,std} = 0.69$
p(P) = 0.73

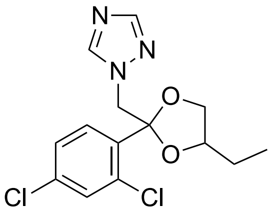

Etaconazole
$\log DT_{50,pred} = 2.42$
$\log DT_{50,std} = 0.59$
p(P) = 0.72

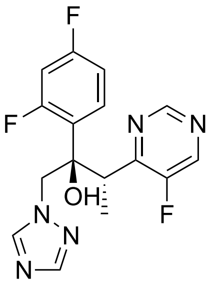

Voriconazole
$\log DT_{50,pred} = 2.45$
$\log DT_{50,std} = 0.69$
p(P) = 0.74

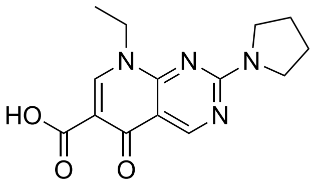

Piromidic Acid
$\log DT_{50,pred} = 2.52$
$\log DT_{50,std} = 0.69$
p(P) = 0.73

Figure S20: **9 out of the 26 ZeroPM substances with a predicted probability of being persistent (p(P)) higher than 70%.** Top: substances with p(P)>80%, bottom: substances with 70% > p(P) > 80%.

References

(S1) Arnot, J.; Gouin, T.; Mackay, D. Development and Application of Models of Chemical Fate in Canada: Practical Methods for Estimating Environmental Biodegradation Rates. 2005.

(S2) Hafner, J.; Fenner, K.; Scheidegger, A. Systematic Handling of Environmental Fate Data for Model DevelopmentIllustrated for the Case of Biodegradation Half-Life Data. *Environmental Science & Technology Letters* **2023**, *10*, 859–864.

(S3) Rasmussen, C. E.; Williams, C. K. I. *Gaussian processes for machine learning*; Adaptive computation and machine learning; MIT Press: Cambridge, Mass, 2006; OCLC: ocm61285753.

(S4) Pedregosa, F.; Varoquaux, G.; Gramfort, A.; Michel, V.; Thirion, B.; Grisel, O.; Blondel, M.; Prettenhofer, P.; Weiss, R.; Dubourg, V.; others Scikit-learn: Machine learning in Python. *Journal of machine learning research* **2011**, *12*, 2825–2830.

(S5) Levi, D.; Gispan, L.; Giladi, N.; Fetaya, E. Evaluating and Calibrating Uncertainty Prediction in Regression Tasks. *Sensors* **2022**, *22*, 5540.

(S6) Yang, C.-I.; Li, Y.-P. Explainable uncertainty quantifications for deep learning-based molecular property prediction. *Journal of Cheminformatics* **2023**, *15*, 13.

(S7) Pernot, P. Properties of the ENCE and other MAD-based calibration metrics. 2023; `http://arxiv.org/abs/2305.11905`, arXiv:2305.11905 [cs].

(S8) Lombardo, A.; Manganaro, A.; Arning, J.; Benfenati, E. Development of new QSAR models for water, sediment, and soil half-life. *Science of The Total Environment* **2022**, *838*, 156004.

(S9) U.S. Environmental Protection Agency Estimation Programs Interface Suite™\ for Microsoft®\ Windows. 2012; Place: Washington, DC, USA.
